# Patterns of sex differences in cancer mortality in Colombia: a population-based analysis, 1980–2023

**DOI:** 10.1016/j.lana.2026.101465

**Published:** 2026-03-30

**Authors:** Oscar Espinosa, Adrian D. Smith, Valeria Bejarano, Gabriela Puentes, Luis-Eduardo Pino, Carolina Wiesner, Esther de Vries, Andrés F. Cardona, Rocco Friebel

**Affiliations:** a Economic Models and Quantitative Methods Research Group, Universidad Nacional de Colombia, Bogotá, D.C., Colombia; b Nuffield Department of Population Health, University of Oxford, Oxford, UK; c Departament of Radiology, Fundación Santa Fe de Bogotá, Bogotá, D.C., Colombia; d OxLER Exponential Medicine & Department of Biomedical Engineering, Universidad de Los Andes, Bogotá, D.C., Colombia; e Instituto Nacional de Cancerología, Bogotá, D.C., Colombia; f Department of Clinical Epidemiology and Biostatistics, Pontificia Universidad Javeriana. Bogotá, D.C., Colombia; g Institute of Research, Science and Education, Luis Carlos Sarmiento Angulo Cancer Treatment and Research Center (CTIC) & Faculty of Medicine, Universidad El Bosque, Bogotá, D.C., Colombia; h Department of Health Policy, The London School of Economics and Political Science, London, UK

**Keywords:** Mortality, Sex, Cancer, Disparities, Colombia

## Abstract

**Background:**

Monitoring long-term trends in cancer mortality is essential to guide prevention and control strategies. In Colombia, where national cancer incidence data are limited, mortality statistics provide a key source of epidemiological insight. We aimed to analyse long-term trends in sex differences in cancer mortality in Colombia between 1980 and 2023, examining variations by cancer type, age group, health insurance scheme, and urban-rural residence.

**Methods:**

We analysed national mortality records (from the DANE) for 1980–2023. Fourteen cancer causes of death were defined using the International Classification of Diseases (ICD-9 and ICD-10). Age-standardised mortality rates per 100,000 population were calculated for individuals aged 20 years or older using Segi's world standard population. Mortality rate ratios (MRRs) were estimated as the ratio of male to female mortality overall and by age group. Years of life lost (YLLs) were calculated assuming a common life expectancy for both sexes.

**Findings:**

Of 274,125 cancer deaths, 54.67% occurred in female individuals and 45.33% in male individuals. Until 1995, female individuals experienced higher mortality, with MR in the 1980s of 87.34 vs. 78.33 (male individuals) and a MRR of 0.90 (95% CI 0.89–0.90). Since 1996, this trend reversed, with MR in the 2020s of 69.23 for female individuals vs. 76.94 for male individuals (MRR: 1.11; 95% CI 1.10–1.12). Over the entire period, female individuals accumulated more YLLs than male individuals, 9.47 million vs. 7.51 million. The most notable sex gaps are observed in bladder (MRR 2.26), oesophageal (MRR 2.18), trachea, bronchus, and lung (MRR 1.72), and stomach cancer (MRR 1.66), with male individuals showing both higher mortality and greater YLL, particularly for stomach cancer. Despite these differences, overall cancer mortality has declined, especially among female individuals aged 50–79.

**Interpretation:**

Long-term mortality trends reveal shifting sex-specific burdens and persistent disparities across cancer types in Colombia. These findings underscore the need for sex-informed prevention and control strategies.

**Funding:**

None.

## Abstract (es)

**Antecedentes:**

El monitoreo de las tendencias de largo plazo en la mortalidad por cáncer es esencial para orientar las estrategias de prevención y control. En Colombia, donde los datos nacionales de incidencia de cáncer son limitados, las estadísticas de mortalidad constituyen una fuente clave de información epidemiológica. El objetivo de este estudio fue analizar las tendencias de largo plazo en las diferencias por sexo en la mortalidad por cáncer en Colombia entre 1980 y 2023, examinando variaciones según tipo de cáncer, grupo de edad, régimen de aseguramiento en salud y residencia urbana-rural.

**Métodos:**

Se analizaron los registros nacionales de mortalidad del DANE para el periodo 1980–2023. Se definieron catorce causas de muerte por cáncer utilizando la Clasificación Internacional de Enfermedades (CIE-9 y CIE-10). Se calcularon tasas de mortalidad estandarizadas por edad por 100,000 habitantes para personas de 20 años o más, utilizando la población estándar mundial de Segi. Las razones de tasas de mortalidad (RTM) se estimaron como el cociente entre la mortalidad masculina y femenina, tanto en general como por grupo de edad. Los años de vida perdidos (AVP) se calcularon asumiendo una esperanza de vida común para ambos sexos.

**Hallazgos:**

De las 274,125 muertes por cáncer, el 54.67% correspondió a mujeres y el 45.33% a hombres. Hasta 1995, las mujeres presentaron mayor mortalidad que los hombres, con una tasa de mortalidad en 1980–1989 de 87.34 en mujeres frente a 78.33 en hombres y una RTM de 0.90 (IC 95%: 0.89–0.90). Desde 1996, esta tendencia se revirtió, con una tasa de mortalidad en 2020–2023 de 69.23 en mujeres frente a 76.94 en hombres (RTM: 1.11; IC 95%: 1.10–1.12). En todo el periodo, las mujeres acumularon más AVP que los hombres: 9.47 millones frente a 7.51 millones. Las brechas por sexo más marcadas se observaron en cáncer de vejiga (RTM 2.26), esófago (RTM 2.18), tráquea, bronquios y pulmón (RTM 1.72) y estómago (RTM 1.66), con mayor mortalidad y más AVP en los hombres, especialmente en cáncer de estómago. A pesar de estas diferencias, la mortalidad general por cáncer ha disminuido, especialmente entre las mujeres de 50–79 años.

**Interpretación:**

Las tendencias de largo plazo en la mortalidad revelan cambios en la carga específica por sexo y desigualdades persistentes entre tipos de cáncer en Colombia. Estos hallazgos subrayan la necesidad de estrategias de prevención y control con enfoque de sexo.

**Financiación:**

Ninguna.

**Research in context**

**Evidence before this study**

We searched PubMed, SciELO, LILACS, and Google Scholar for articles and official reports published between Jan 1, 1980, and Dec 31, 2025, using combinations of the terms “cancer mortality”, “cancer epidemiology”, “years of life lost”, “sex differences”, “Latin America”, and “Colombia”. Global initiatives such as GLOBOCAN provide critical point-in-time estimates of cancer incidence and mortality, while national reports from the *Instituto Nacional de Cancerología* of Colombia describe epidemiological patterns and outline health system challenges and strategic priorities. Regional studies have documented ongoing cancer transitions in Latin America, and emerging Colombian research has explored inequalities across selected subpopulations, including by migration status and ethnicity. However, the available evidence remains fragmented. These documents do not present long-term, disaggregated mortality trends spanning multiple decades, and most subpopulation studies rely on cross-sectional estimates or short observation periods that mask how long-term cancer mortality and years of life lost trends differ by sex, cancer type, and age group.

**Added value of this study**

This study is the first to present a four-decade analysis (1980–2023) of sex differences in cancer mortality and years of life lost in Colombia, stratified by age group, tumour type, insurance scheme, and urban-rural area. Using official national mortality data, we document a historical shift from higher female cancer mortality in the 1980s to a predominance in men since the early 2000s, while women continue to account for a greater share of YLLs. Our findings reveal persistent sex disparities in cancer mortality, particularly among individuals in the contributory insurance scheme -that is, those formally employed- and in urban areas. We also identify tumour types with disproportionately high male mortality (such as bladder, oesophageal, and stomach cancers) highlighting the need for sex-specific preventive and clinical strategies. This evidence provides a critical foundation to inform more equitable and effective cancer control policies in Colombia.

**Implications of all the available evidence**

The increasing cancer burden in Colombia, combined with persistent sex disparities in mortality and premature death, underscores structural limitations in early detection, treatment access, and preventive strategies. Compared to high-income countries, cancer outcomes in Colombia remain suboptimal, especially among populations with fewer resources or delayed care. Incorporating sex-sensitive epidemiological insights into national cancer control efforts is essential to improve equity, guide health system planning, and reduce avoidable cancer deaths.

## Introduction

Cancer remains a major contributor to global mortality, with sex-specific differences consistently observed in both incidence and survival.^1^, ^2^, ^3^, ^4^ In 2022, an estimated 20 million new cancer cases were reported worldwide alongside 9.7 million cancer-related deaths. Current estimates indicate that approximately 1 in 5 individuals will develop cancer during their lifetime, while about 1 in 9 men and 1 in 12 women will die from the disease, reflecting its substantial global burden.^5^ Evidence suggests that, for most cancer types, being male is associated with both a higher risk of cancer and worse survival outcomes.^6^, ^7^, ^8^, ^9^ These disparities are shaped by complex interactions between biological mechanisms (such as hormonal influences and genetic susceptibility) and socio-behavioural factors including smoking, alcohol use, diet, high body mass index, occupational exposures, and delays in healthcare seeking.^10^, ^11^, ^12^

Trends in cancer mortality provide information on the quality of health systems and social responses to this phenomenon; the analysis of sex differences in mortality trends reveals variations according to sociopolitical and healthcare contexts within a sex perspective. Latin America presents a particular epidemiological setting, characterised by a mixed-race population of European, Black, and Indigenous origin in the process of rapid urbanisation and industrialisation and with an increasing cancer burden and inequalities in detection and treatment.^13^^,^^14^

Within this region, Colombia has experienced remarkable changes in its demographic structure and health system over the past four decades. The introduction of a healthcare model under a managed competition approach,^15^ comprising contributory (formally employed) and subsidised (vulnerable populations) insurance schemes, has improved coverage and access but also introduced structural inequities.^16^ These disparities and genetic ancestries may differentially affect men and women, especially in rural and socioeconomically disadvantaged populations, where access to timely cancer diagnosis and treatment remains limited.^17^

Sex differences in cancer outcomes have been reported globally,^4^^,^^11^ and national cancer-specific mortality analyses stratified by sex have been conducted in Colombia.^18^, ^19^, ^20^, ^21^, ^22^ However, little is known about how these sex-based patterns have evolved over the past 4 decades, during which systemic reforms and strong demographic transitions occurred.

In this study, we analysed national cancer mortality data from 1980 to 2023, covering 14 major cancer types in Colombia, including both sex-specific (e.g., breast, prostate, cervical) and non-sex-specific malignancies (e.g., lung, stomach, colorectal). Using national cause-of-death statistics, we characterised sex-specific mortality trends over time and described inequalities related to health insurance scheme and urban-rural area. The study was conducted and reported in accordance with the STROBE and SAGER guidelines to ensure transparency, reproducibility, and appropriate consideration of sex-based differences.^23^^,^^24^

## Methods

### Data sources

We utilised mortality records from the Colombian National Administrative Department of Statistics (DANE) for the period between 1980 and 2023. DANE documents non-foetal deaths based on certificates issued by physicians, qualified health professionals, or forensic pathologists.^25^ The validity of DANE's mortality registry has been rigorously assessed and classified as high-quality according to international standards.^26^ Furthermore, formal validation studies confirm that Colombia's vital statistics system meets the criteria for reliable cause-of-death analysis, distinguishing real epidemiological trends from data artifacts.^27^

Population denominators were derived from DANE's official demographic projections, based on census data.^28^ These age- and sex-specific population projections were used to calculate mortality rates for the following groups: 20–29 years, 30–39 years, 40–49 years, 50–59 years, 60–69 years, 70–79 years, and ≥80 years. Data on health insurance affiliation were obtained from the Single Database of Affiliates (BDUA), which distinguishes between the contributory and subsidised schemes. BDUA data were available from 2012 onwards.

### Mortality data

We analysed overall deaths due to any cancer and 14 categories of specific cancers (Supplementary Table S1, p 1). These categories were selected based on three criteria: (i) high disease burden, accounting for approximately 70% of total cancer mortality in Colombia; (ii) coding stability, ensuring robust comparability across the transition from ICD-9 to ICD-10; and (iii) relevance to sex-based analysis, encompassing both sex-specific malignancies and non-sex-specific cancers associated with modifiable risk factors. The categories were aligned with the International Classification of Diseases–ICD-9 version 1977 (1980–1996) and ICD-10 version 2019 (1997–2023). Although ICD-11 was officially presented in 2019, its adoption in Colombia was formally established by the Ministry of Health and Social Protection through Resolution 1442 of 2024,^29^ which includes a 12-month transition period. Consequently, ICD-11 was not in operational use for mortality registration during the study period from 2019 to 2023. For cervical cancer, we redistributed the deaths coded with cause “uterus Not Otherwise Specified–NOS” proportionally between cervix and corpus uteri, in line with international standards.^30^ Urban-rural classification was included from 1985 onward, using the Colombian National Planning Department's criteria.^31^ Records with causes not fitting the defined categories, missing location (<0.003% of cases), or lacking health insurance scheme information (∼2.6%) were excluded in the correspondent analysis.

### Statistical approach

All mortality rates (MRs) were expressed per 100,000 population and age-standardised using Segi's world population as the reference.^32^ Mortality rate ratios (MRRs) were computed as the ratio of male to female mortality, both overall and within specific age strata, for the 1980–2023 period. Years of life lost (YLLs) were estimated using a modified version of the method proposed by Espinosa et al.,^33^ assuming a common life expectancy for both sexes (per rate per 100,000 inhabitants). The life expectancy at birth and across quinquennial age groups of the Colombian population was calculated using the Lee-Carter model (Supplementary Table S2, p 2),^34^ thus the corresponding formula reduces to:

$$YLL_{t,s,cc}=\sum_{i\in age\;group}d_{i,t,s,cc}*\left(life\;expectancy_{t}-AIRP_{i}\right)\text{;}\;if\;{life\;expectacy}_{t}>AIRP_{i}\text{,}$$

where *t* denotes the year, *s* the sex, and *cc* the cancer cause of death. The term *d* represents the number of deaths in age group *i*, year *t*, sex *s*, and cancer cause *cc*. *AIRP* denotes the age interval midpoint corresponding to age group *i*. Age groups are defined as decadal age intervals, extending up to 80 years or older.

Wald confidence limits under a Poisson model assumption, this is MR∗*exp*(±1.96∗*A*^−1/2^) where *A* is the number of the age-standardise deaths; were employed to compute 95% confidence intervals (CI) for MRs. CI for MRRs were obtained by first estimating the CI for the numerator and denominator rates separately and then deriving it from the ratio of their corresponding upper and lower bounds.

An additional analysis regarding the age distribution of cancer mortality was conducted. Percentages were calculated descriptively, as the proportion of deaths in each age group relative to the total number of deaths observed for a given cancer cause, sex, and year. All analyses were conducted using R software (version 4.4.3). As the study used anonymised, publicly accessible data, ethical approval was not required.

### Role of the funding source

None.

## Results

**Fig. 1:**
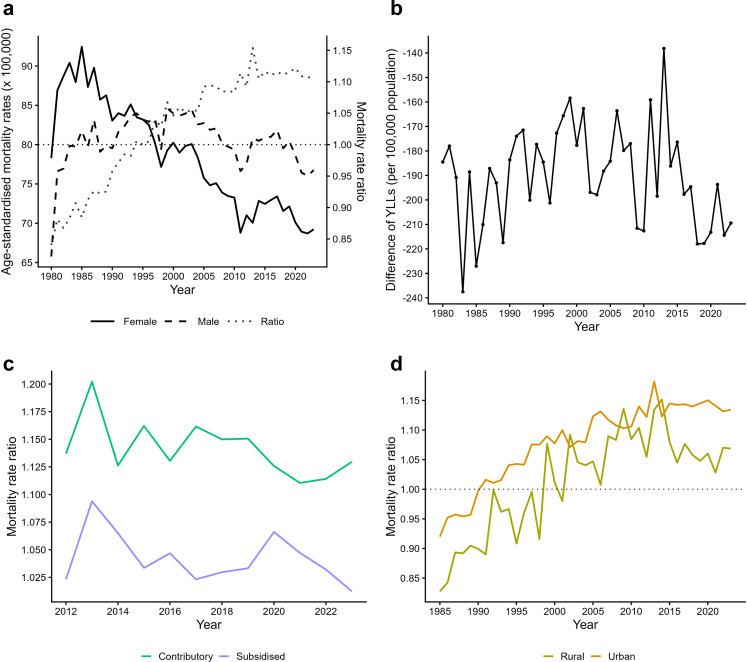
**(a)** Age-standardised all neoplasms mortality rates for female and male overall, (right axis mortality rate ratio). **(b)** Difference of years of life lost (YLLs) per 100,000 population (male–female). **(c)** Cancer mortality rate ratios between sexes by health insurance scheme. **(d)** Cancer mortality rate ratios by urban and rural area (1980–2023).

A total of 274,125 deaths due to neoplasms were identified between 1980 and 2023. Cancer mortality behaviour changed over time, as shown in Fig. 1a. Until 1995 female individuals experienced higher cancer mortality than male individuals: between 1980 and 1989 female individuals had a MR of 87.34 deaths/100,000 inhabitants while male individuals had a MR of 78.33 deaths/100,000 inhabitants with a MRR of 0.90 (95% CI 0.89–0.90). Female individuals accumulated more YLLs than male individuals, 1.29 million vs. 0.98 million (see Table 1). However, from the mid-1990s onward, male mortality began to surpass female mortality, giving that from 1996 male individuals experienced higher mortality, with the gap growing from 1% between 1990 and 1999 (MRR: 1.01; 95% CI 1.01–1.01) to 11% during 2020–2023 (MRR: 1.11; 95% CI 1.10–1.12). Throughout the entire period, estimates of annual life years lost to cancer were consistently higher for female individuals than male individuals. The difference peaked in 1983, with 237/100,000 inhabitants greater YLL for women vs. men, reducing to 138/100,000 inhabitants in 2013 and increasing again to 209/100,000 by 2023 (Fig. 1b).

**Table 1:** Burden of cancers in female and male of Colombia by decade between the 1980s and the 2020s.

Decade	Female		Male		Comparison	
	MR/100,000 pop	Years of life lost (millions)	MR /100,000 pop	Years of life lost (millions)	Difference in MR^a^/100,000 pop	MRR^b^ ratio
**All people**						
1980–2023	75.85	9.47	80.25	7.51	4.41	1.06 (1.05, 1.06)
1980–1989	87.34	1.29	78.33	0.98	−9.01	0.90 (0.89, 0.91)
1990–1999	81.93	1.60	82.44	1.24	0.51	1.01 (1.01, 1.01)
2000–2009	76.77	2.21	82.37	1.75	5.60	1.07 (1.06, 1.08)
2010–2019	71.79	3.20	79.92	2.64	8.12	1.11 (1.10, 1.13)
2020–2023	69.23	1.33	76.94	1.07	7.71	1.11 (1.10, 1.12)
**Contributory health insurance**						
2010–2019	68.63	1.21	79.04	0.99	10.41	1.15 (1.13, 1.17)
2020–2023	63.95	0.60	71.62	0.48	7.66	1.12 (1.10, 1.14)
**Subsidised health insurance**						
2010–2019	71.27	1.25	74.25	1.01	2.99	1.04 (1.04, 1.05)
2020–2023	67.73	0.66	70.35	0.53	2.62	1.04 (1.03, 1.04)

95% CIs in parentheses. MR stratified by health insurance scheme are slightly lower than the total MR because deaths with missing insurance information are excluded from scheme-specific estimates, whereas they are included in the total population rate.

a A positive number means more mortality rates (MR) in males.

b MRR (mortality rate ratio; a number >1 means more mortality in males).

Fig. 1c displays trends in cancer mortality rate ratios by health insurance scheme. The sex gap, with higher male mortality exists in both schemes, but was wider in the contributory scheme (2020–2023 MRR: 1.12; 95% CI 1.10–1.14) vs. the subsidised scheme (2020–2023 MRR: 1.04; 95% CI 1.03–1.04) (see Table 1). Despite lower mortality rates, female individuals in the subsidised scheme had higher YLL; 660,000 more in the 2020s, compared to 600,000 more in the contributory scheme. Supplementary Figure S1 (p 5) highlights liver, skin, and colorectal cancers as the most sex-disparate in the contributory scheme compared to the subsidised with MRR of 1.45 (95% CI 1.39–1.51) vs. 1.00 (95% CI 0.98–1.02); 1.65 (95% CI 1.37–1.99) vs. 1.00 (95% CI 0.93–1.06) and 1.20 (95% CI 1.18–1.22) vs. 1.03 (95% CI 1.02–1.04), respectively by 2023.

Table 2 summarises the sex gap by cancer type for the 1980–2023 period. The largest gaps are observed in bladder (MRR 2.28; 95% CI 0.93–5.62), oesophageal (MRR 2.22; 95% CI 1.18–4.16), trachea, bronchus and lung (MRR 1.74; 95% CI 1.44–2.10) and stomach cancer (MRR 1.71; 95% CI 1.46–2.00), with male individuals showing both higher mortality and greater YLL, particularly for stomach cancer (1,390,000 more YLL). In contrast, pancreatic cancer presents equal mortality (MRR 1.00; 95% CI 1.00–1.01) but higher YLL in male individuals (10,000 more). Slightly higher mortality was observed in female individuals for cancer in colon, rectum and anal canal carcinomas (female MR 5.46 vs. male MR 5.44, MRR 1.00; 95% CI 1.00–1.00).

**Table 2:** Cancer-specific mortality and years of life lost in Colombia, by sex, between the 1980 and the 2023.

Type of cancer	Female		Male		Comparison
	MR^a^/100,000 pop	Years of life lost (millions)	MR/100,000 pop	Years of life lost (millions)	MRR^b^ ratio
All neoplasms	75.85	9.47	80.25	7.51	1.06 (1.05, 1.06)
Bladder	0.54	0.04	1.23	0.07	2.28 (0.93, 5.62)
Breast	9.47	1.46	–	–	–
Cervical	9.02	1.55	–	–	–
Colon, rectum and anal canal	5.46	0.57	5.44	0.53	1.00 (1.00, 1.00)
Oesophagus	1.04	0.08	2.31	0.17	2.22 (1.18, 4.16)
Leukaemia	2.34	0.45	2.90	0.53	1.24 (1.09, 1.41)
Liver	2.99	0.27	3.24	0.28	1.08 (1.04, 1.13)
Ovary	3.10	0.45	–	–	–
Pancreas	2.97	0.26	2.98	0.27	1.00 (1.00, 1.01)
Prostate	–	–	10.16	0.37	–
Skin	0.45	0.06	0.57	0.06	1.26 (0.92, 1.73)
Stomach	8.46	0.92	14.44	1.39	1.71 (1.46, 2.00)
Trachea, bronchus, lung	6.17	0.57	10.71	0.84	1.74 (1.44, 2.10)
Uterine	1.18	0.12	–	–	–

95% CIs in parentheses.

a MR (standardised mortality rate).

b MRR (mortality rate ratio; a number >1 means more mortality in males).

**Fig. 2:**
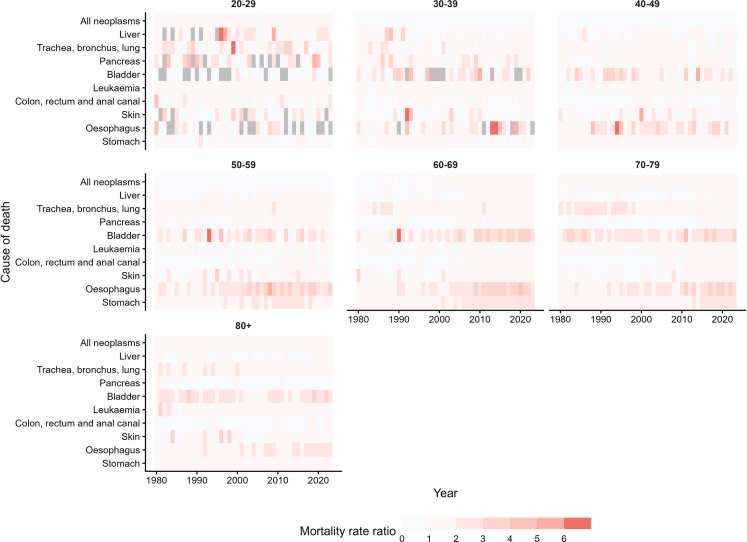
Mortality rate ratios in age-specific mortality by type of cancer in Colombia, by age group between 1980 and 2023. Note: grey values indicate no recorded mortality for females, but mortality was recorded for males. Mortality rate ratio >1 means higher male mortality.

Fig. 2 and Supplementary Table S3 (p 3) explore the age decomposition of cancer mortality. In the 20–29 age group, male mortality exceeds female mortality (in some cases, females recorded zero deaths), particularly for oesophageal (2020–2023 MRR 2.49; 95% CI 1.50–4.15), skin (2020–2023 MRR 2.14; 95% CI 1.69–2.70) and liver cancers (2020–2023 MRR 2.08; 95% CI 1.75–2.47). For ages 30–39, female mortality surpasses male mortality, recently in the 2020s by 44% (MRR 0.69; 95% CI 0.69–0.70), particularly for skin cancer (MRR 0.89; 95% CI 0.87–0.91), although in the remaining causes in earlier decades, male rates were higher. A similar trend is observed for ages 40–49, especially in colon, rectum, and anal canal carcinomas (2010–2019 MRR 0.97; 95% CI 0.97–0.97). Among those aged 50–59, the sex gap begins to widen, particularly for oesophagus cancer, reaching over 206% more mortality between 2000 and 2009 (MRR 3.06; 95% CI 2.90–3.23).

For individuals aged 60–69 and 70–79, overall cancer mortality in male individuals surpassed female individuals, particularly in stomach (MRR 2.35; 95% CI 2.32–2.39 and MRR 2.26; 95% CI 2.24–2.29), oesophageal (MRR 3.28; 95% CI 3.09–3.49 and MRR 3.06; 95% CI 2.92–3.20) and bladder cancers (MRR 3.09; 95% CI 2.89–3.31 and MRR 2.76; 95% CI 2.64–2.88) by the 2020s. In individuals aged 80 or more, while male mortality remains higher overall, female mortality surpassed males for pancreatic (2020–2023 MRR 0.85; 95% CI 0.83–0.86) and colon, rectum and, anal canal carcinomas (2010–2019 MRR 0.96; 95% CI 0.95–0.96).

Despite these differences, overall cancer mortality declined over time, especially among female individuals aged 50–79 (Supplementary Figure S2, p 6). The age distribution by cancer type is detailed in Supplementary Figures S3 and S4 (pp 7–8), where the 80+ age group shows increases from 1980 to 2023 up to 20% participation (male bladder cancer). There was a steady increase over the entire period (1980–2023) in the share of skin cancer among individuals aged 40–49, rising from 4% to 8% in female individuals and from 7% to 11% in male individuals. Stomach cancer among female individuals also showed increased representation, rising from 3% to 5% in the 30–39 age group, and from 9% to 11% in the 40–49 age group. Cervical cancer among women aged 30–39 increased by 2%. Other cancers exhibited declining trends; there was a 16% decrease in liver cancer among male individuals aged 60–69, a 12% decrease in leukaemia among both sexes aged 20–29, a 10% decrease in uterine cancer at ages 40–49, an 8% decline in both uterine and ovarian cancer (ages 50–59), and a 7% decrease in breast cancer among female individuals aged 40–49.

Fig. 1d shows the urban–rural trends. In urban areas, female cancer mortality exceeded that of males until 1990; in rural areas, this persisted until 1998. Since then, the gap has widened in urban zones, reaching an MRR of 1.13 (95% CI 1.12–1.15) in 2023, while the rural gap is narrower (MRR 1.07; 95% CI 1.06–1.08). Supplementary Figure S5 (p 9) presents departmental variations, showing higher female mortality in the 1980–1989 period although higher values of male mortality can be seen in San Andrés (MRR 1.52; 95% CI 1.45–1.59), Chocó (MRR 1.22; 95% CI 1.19–1.25), and La Guajira (MRR 1.48; 95% CI 1.37–1.60). By 1990–1999, male mortality began to exceed female mortality in more departments, including remote ones such as Amazonas (MRR 1.23; 95% CI 1.20–1.26), Vaupés (MRR 1.76; 95% CI 1.58–1.96), and Guaviare (MRR 1.27; 95% CI 1.24–1.32). As seen in the national level, male mortality predominated from the 2000s onwards, although in some departments this trend reversed, with higher female mortality re-emerging, particularly in the 2020s: Amazonas (MRR 0.73; 95% CI 0.70–0.76), Vaupes (MRR 0.77; 95% CI 0.74–0.80), Guainía (MRR 0.82; 95% CI 0.80–0.84), Vichada (MRR 0.90; 95% CI 0.89–0.92) and Guaviare (MRR 0.85; 95% CI 0.83–0.87).

Finally, Supplementary Figure S6 (p 10) disaggregates trends by age and cancer type across urban-rural zones. Before age 40, gaps are similar across regions. From age 40 onwards, disparities between sexes grow, especially for oesophageal (40–49 average MRR 1.88 rural vs. 2.12 urban), stomach (50–59 average MRR 1.66 rural vs. 1.86 urban), and trachea, bronchus, and lung cancers (40–49 average MRR 1.17 rural vs. 1.34 urban) in urban zones. In later decades of life (ages 50–80+), bladder cancer contributes substantially to the growing gap, particularly in urban populations, with average MRRs of 2.11 in urban areas vs. 1.56 in rural areas among those aged 50–59, 2.51 vs. 1.84 (70–79), and 2.35 vs. 2.08 (80+).

## Discussion

This detailed analysis of cancer mortality trends in Colombia over four decades reveals persistent sex disparities, with male individuals exhibiting consistently higher mortality rates for most cancer types as of the late 1990s, particularly for those cancer types associated with tobacco use and occupational exposures such as lung, oesophageal, and bladder cancers. These findings highlight the complex interplay between biological, behavioural, and social determinants of cancer outcomes within the context of Colombia's evolving demographic and healthcare landscape. The differing patterns observed by insurance scheme and urban-rural residence may reflect such behavioural determinants but also suggest structural inequities in access to timely diagnosis and treatment, which may have contributed to the observed mortality disparities.

Absolute levels of cancer mortality in recent years have fallen to the lowest ranges of cancer mortality worldwide, comparable to those in the United States, Peru and Ecuador. These outcomes compare positively to neighbouring countries such as Peru which has lower cancer incidence but comparable mortality.^3^^,^^5^ Similarities to high-income settings such as the United States are more complex; the US has a higher cancer incidence, implying that survival probabilities from diagnosis in Colombia are comparatively poor, or that diagnosis occurs later. Cancer incidence in the United States (US) does show variation by sex, with a general tendency for higher incidence among male individuals, more than 17-fold in the US Latino population, but between 1.4 and 4 times for other ethnicities.^35^ Sex-based differences in incidence are reflected by similar or even greater differences in mortality, since for most cancers that occur in both sexes, prognosis tends to be better among female individuals.^36^^,^^37^

Colombian cancer mortality data for 1980 to 2023 reveal a trend until 1994 of higher female mortality that slowly shifts towards higher male mortality from 1996. However, over the entire period the female excess in YLL remains.^38^^,^^39^ This apparent paradox can be explained by differences in the cancer types contributing to mortality. Cervical cancer is a major cause of death among women and tends to occur at younger ages: over 50% of cervical cancer deaths occur between ages 30 and 59. In contrast, more than 70% of prostate cancer deaths occur in men aged over 70. Consequently, the loss of a 35-year-old mother's life represents far more potential years than the death of an octogenarian man from prostate cancer.^40^ These patterns highlight the need to look beyond crude mortality rates and examine trends over time, by health scheme, urban-rural area, and age.

Related to the temporal shift in the sex gap, three interacting factors appear to be decisive. First, male smoking and heavy drinking remained high through the 1990s, fuelling a delayed wave of lung, upper-gastrointestinal and liver cancers.^41^ Secondly, the systematic roll-out of cervical cytology, followed by Human Papillomavirus (HPV) testing and vaccination, alongside declining fertility rates (a known cofactor for cervical carcinogenesis), contributed to reduced cervical cancer mortality.^41^^,^^42^ Thirdly, broader gains in obstetric, cardiovascular, and infectious-disease control extended female life expectancy, increasing the likelihood of developing cancer and shifting deaths to older ages, although this did not fully close the YLL gap.^43^

The sex differential in cancer mortality is considerably wider in the contributory health insurance scheme than in the subsidised scheme (MRR in the 2020–2023: 1.12 vs. 1.04). Part of this excess may be artefactual: higher diagnostic intensity among men insured under the contributory scheme may increase the likelihood that cancers are detected and recorded as the underlying cause of death, particularly for prostate and renal cancers. By contrast, women still incur more YLL, reflecting delayed diagnosis and suboptimal treatment. HPV vaccination, screening and early cancer detection with trained and empowered general practitioners (GPs) promoting targeted testing packages that combine multidisciplinary and integrated treatment delivery could eliminate this avoidable shortfall and harmonise outcomes across schemes.^44^^,^^45^

Bladder, oesophageal, lung and stomach cancers display the largest male excesses (MRR ≥1.71), mirroring well-known exposures and therefore cancer incidence:^5^ smoking, alcohol, occupational carcinogens and *Helicobacter pylori* infection. Fortunately, apart from bladder cancer all these very aggressive cancer types showed an overall decreasing trend. Stomach cancer alone accounts for an extra 467,330 male YLL, underscoring the need for population-wide *H. pylori* eradication and salt-reduction policies. By contrast, pancreatic cancer shows equal mortality yet is increasing and causes more YLL for men, suggesting biological differences in causes, molecular biology and post-diagnostic survival.^40^ Notably, colon and rectal cancers now impose a slightly higher burden on women, consistent with rising obesity and sedentary behaviour in the female workforce.^46^ Skin cancer presents a complex picture: absolute rates are rising in both sexes, but more rapidly among men in high-altitude regions and areas with greater ultraviolet radiation exposure, emphasising the need for occupational sun-protection programs.^47^ Conversely, cervical cancer remains the single most considerable contributor to female YLL in the subsidised scheme, highlighting the unfinished agenda of universal HPV vaccination, screening, treatment of high-risk preneoplastic lesions and opportune and high-quality treatment of those with invasive cancers.^44^^,^^48^

Age decomposition paints a nuanced picture. Among 20–29-year-olds, men carried a greater risk, albeit on small numbers, for oesophageal, liver and skin cancers, possibly linked to hereditary conditions, perinatal hepatitis-B infection, early harmful drinking and differences in hormonal protective factors.^49^ Between 30- and 49-year-old female mortality rises steeply; by the 2020s, women in their thirties died 44.38% more often from cancer than men, driven by cervical, breast, and increasingly colorectal cancers. After the age of 50, where opportunistic breast cancer screening is advised, the advantage reverses: in men aged 60–79, stomach, oesophageal, and bladder cancers push MRRs above two.^50^ At 80+ years, male mortality still predominates overall. However, female deaths from pancreatic and colorectal cancers slightly exceed male rates, echoing global trends towards feminisation of metabolic cancers in very old age.^3^^,^^46^

Urban–rural stratification exposes divergent trajectories in the sex gap for cancer mortality. Female rates exceeded male rates nationwide until the early-1990s, yet the inflection point came six years earlier in cities than in the countryside. Thereafter urban areas have drifted apart: by 2023 men in metropolitan centres died 13% more often from cancer than women (MRR 1.13, 95% CI 1.12–1.15). The few departments where female mortality surpassed male mortality in the 2020s are Amazonas, Vaupés, Guainía, Vichada, and Guaviare, predominantly remote rural territories marked by high poverty and scant health-service reach, mainly due to infection-associated cancers. These pockets of female excess suggest that low HPV vaccination, delayed diagnosis, poor access to treatment and unmitigated infectious hazards continue to be very important, underscoring the need for targeted cancer-control strategies attuned to extreme socioeconomic deprivation and geographic remoteness.^51^

Male smoking prevalence exceeded 30% until the mid-2000s, double that of women, accounting for much of the lung and bladder excess.^52^ Alcohol-attributable fractions remain higher in men, aligning with oesophageal and liver gaps.^53^ Conversely, incomplete HPV vaccination outside Bogotá and heterogeneous mammography quality and varying use of opportunistic screening opportunities impede faster female gains.^48^^,^^54^ Rising obesity and diabetes in both sexes threaten to blunt progress, particularly for colorectal and pancreatic cancers. Policy action on diet, physical activity and glycaemic control and improving uptake of existing opportunistic screening for colorectal cancer, which has very poor survival compared to other countries, is therefore indispensable.^55^^,^^56^

These findings carry clear public-health implications. Policies should prioritise uptake of colorectal screening, smoking cessation, *H. pylori* screening and hepatitis-B vaccination, especially among middle-aged men where the burden is highest, while simultaneously expanding HPV or other cervical-screening outreach and high coverage of HPV catch-up vaccination. In addition, health-service planners must bolster GP diagnostic capacity in remote areas by ensuring rapid access to colposcopy, endoscopy, quality-assured breast imaging and timely pathology services, leveraging telepathology and artificial-intelligence tools where feasible to help close the YLL gap.^57^

This study has several limitations that should be considered when interpreting the findings. First, our study is descriptive by design and does not incorporate information on individual-level risk factors such as tobacco use, occupational exposures, comorbidities, or health-seeking behaviours. Nor does it adjust for potential confounders beyond age. Further research, employing longitudinal or quasi-experimental methods (particularly those capable of incorporating behavioural, biological, and structural determinants), is necessary to understand better the mechanisms driving the observed sex and urban-rural disparities in cancer mortality.

Second, Colombia's vital registration system has improved substantially over the last four decades, positioning it as a reference model for low- and middle-income countries (especially in cancer);^26^^,^^27^ however, earlier records (particularly from the 1980s and early 1990s) may be affected by incomplete reporting or cause-of-death misclassification. This may have caused over- or underestimations of the true mortality rates, particularly in the earlier periods.^20^^,^^22^ These limitations are more likely to occur in rural or historically underserved regions, where diagnostic capacity and certification practices were and remain more limited. Under-registration may have led to regional or temporal underestimation of cancer mortality, especially for certain tumour types with historically low detection rates (notably, for cervical cancer, a correction was applied once this issue was identified).

Third, the analysis of disparities by health insurance scheme was constrained to the years following the availability of BDUA data (2012 onward). As such, we could not assess how earlier phases of health system reform may have influenced access to timely diagnosis or treatment across the full 44-year period. Nonetheless, including scheme-specific data from recent years adds valuable granularity and highlights emerging inequities within the restructured health system.

Fourth, the lack of systematically recorded ethnicity data before 2018 prevented an intersectional analysis of cancer mortality disparities by ethnic-racial group, despite ethnicity being a key health determinant in Colombia.^58^

This study described long-term patterns in sex-specific cancer mortality in Colombia but also exposes critical blind spots that demand deeper inquiry. Several lines of research are especially urgent. Population-based surveys to measure the exposure to tobacco and air pollution, dietary habits and sedentary behaviour are in urgent need of updating. The biological interface between sex hormones, metabolic risk, and tumour progression -particularly for colorectal and pancreatic cancers-requires large-scale prospective cohorts with integrated biomarker data. Policy scenarios could be developed to inform decision-making: under constrained financing, which intervention package yields greater population impact–male-focused tobacco, or expanded cervical screening, perhaps with self-tests in remote areas, and HPV catch-up among underserved women? Comparative effectiveness trials stratified by insurance scheme could offer actionable answers.

Finally, the persistence of sex disparities despite nominal service coverage calls for qualitative and mixed-methods studies on sex norms, stigma, and help-seeking behaviours in remote and disadvantaged settings. By consolidating national mortality trends across cancer types, insurance schemes, age groups, and regions over four decades, our study provides the foundational epidemiological platform on which these deeper investigations can now build. It would be important to carry out a study in regions where long-standing cancer incidence data are available from cancer registries, to compare incidence, survival and mortality trends to better understand and interpret observed trends.

## Conclusion

Colombia has transitioned from a pattern of female-dominated cancer mortality in the 1980s to a male-dominated one today, yet women continue to lose more potential life years. Disparities vary by age, tumour type, insurance scheme and geography, reflecting a mosaic of biological, behavioural and systemic determinants. A dual agenda is therefore required: aggressive primary prevention targeting male-linked exposures, and, for women, equitable measures such as HPV vaccination, along with timely diagnosis and treatment for female cancers, especially in deprived settings. Embedding these priorities into the country's next decennial cancer plan is critical if Colombia is to achieve both efficiency and fairness in cancer outcomes.

## Contributors

OE: conceptualisation, data curation, formal analysis, investigation, methodology, project administration, resources, software, supervision, validation, visualisation, writing—original draft, and writing—review & editing.

AS: formal analysis, investigation, methodology, supervision, validation, and writing—review & editing.

VB: data curation, formal analysis, investigation, resources, software, validation, visualisation, writing—original draft, and writing—review & editing.

GP, LEP, CW, EV, AFC: formal analysis, investigation, validation, and writing—review & editing.

RF: formal analysis, investigation, resources, validation, and writing—review & editing.

OE and VB directly accessed and verified the underlying data reported in the manuscript. All authors are responsible for the decision to submit the manuscript.

## Data sharing statement

The data and programming code are available at https://github.com/oaespinosaa/Mortality_by_sex_cancer_Colombia.

## Disclosure of AI or AI-assisted technologies

The authors used OpenAI GPT o3 exclusively for English grammar and language editing.

## Declaration of interests

We declare no competing interests.

## References

[1] Bray F., Laversanne M., Weiderpass E., Soerjomataram I. (2021). The ever-increasing importance of cancer as a leading cause of premature death worldwide. Cancer.

[2] Zhu Y., Shao X., Wang X., Liu L., Liang H. (2019). Sex disparities in cancer. Cancer Lett.

[3] Siegel R., Kratzer T., Giaquinto A., Sung H., Jemal A. (2025). Cancer statistics, 2025. CA Cancer J Clin.

[4] Kocarnik J.M., Compton K., Global Burden of Disease 2019 Cancer Collaboration (2022). Cancer incidence, mortality, years of life lost, years lived with disability, and disability-adjusted life years for 29 cancer groups from 2010 to 2019. JAMA Oncol.

[5] Bray F., Laversanne M., Sung H. (2024). Global cancer statistics 2022: GLOBOCAN estimates of incidence and mortality worldwide for 36 cancers in 185 countries. CA Cancer J Clin.

[6] Radkiewicz C., Johansson A., Dickman P., Lambe M., Edgren G. (2017). Sex differences in cancer risk and survival: a Swedish cohort study. Eur J Cancer.

[7] Cook M., McGlynn K., Devesa S., Freedman N., Anderson W. (2011). Sex disparities in cancer mortality and survival. Cancer Epidemiol Biomarkers Prev.

[8] Najari B., Rink M., Li P. (2013). Sex disparities in cancer mortality: the risks of being a man in the United States. J Urol.

[9] Kim H.-I., Lim H., Moon A. (2018). Sex differences in cancer: epidemiology, genetics and therapy. Biomol Ther (Seoul).

[10] Edgren G., Liang L., Adami H.-O., Chang E. (2012). Enigmatic sex disparities in cancer incidence. Eur J Epidemiol.

[11] Tran K., Lang J., Compton K. (2022). The global burden of cancer attributable to risk factors, 2010–19: a systematic analysis for the Global Burden of Disease Study 2019. Lancet.

[12] Jackson S., Marks M., Katki H. (2022). Sex disparities in the incidence of 21 cancer types: quantification of the contribution of risk factors. Cancer.

[13] Piñeros M., Laversanne M., Barrios E. (2022). An updated profile of the cancer burden, patterns and trends in Latin America and the Caribbean. Lancet Reg Health Am.

[14] Goss P., Lee B., Badovinac-Crnjevic T. (2013). Planning cancer control in Latin America and the Caribbean. Lancet Oncol.

[15] Castano R., Prada S., Maldonado N., Soto V. (2024). Managed competition in Colombia: convergence of public and private insurance and delivery. Heal Econ Policy Law.

[16] Mora-Moreo L., Estrada-Orozco K., Espinosa O., Melgarejo L. (2023). Characterization of the population affiliated to the subsidized health insurance scheme in Colombia: a systematic review and meta-analysis. Int J Equity Health.

[17] de Vries E., Buitrago G., Quitian H., Wiesner C., Castillo J. (2018). Access to cancer care in Colombia, a middle-income country with universal health coverage. J Cancer Policy.

[18] Barreto C., Limas L., Porras A., Rico A. (2023). The burden of gastric cancer disease from 2010 to 2019 in Tunja, Boyacá, Colombia. Rev Colomb Gastroenterol.

[19] Arias-Ortiz N., Rodríguez-Betancourt J. (2022). Trends in cancer incidence and mortality in Manizales, Colombia, 2008-2017. Colomb Méd.

[20] Pardo C., Cendales R. (2018). Cancer incidence estimates and mortality for the top five cancer in Colombia, 2007-2011. Colomb Méd.

[21] Navarro E., Caballero H., Cortés A. (2024). https://www.infocancer.co.

[22] Pardo C., de Vries E., Buitrago L., Gamboa O. (2017).

[23] Heidari S., Babor T., De Castro P., Tort S., Curno M. (2016). Sex and gender equity in research: rationale for the SAGER guidelines and recommended use. Res Integr Peer Rev.

[24] von Elm E., Altman D., Egger M., Pocock S., Gøtzsche P., Vandenbroucke J. (2007). The Strengthening the Reporting of Observational Studies in Epidemiology (STROBE) statement: guidelines for reporting observational studies. Lancet.

[25] Departamento Administrativo Nacional de Estadística (2025). Estadísticas vitales. https://www.dane.gov.co/index.php/estadisticas-por-tema?view=category&id=33.

[26] Mikkelsen L., Phillips D., AbouZahr C. (2015). A global assessment of civil registration and vital statistics systems: monitoring data quality and progress. Lancet.

[27] Cendales R., Pardo C. (2022). Calidad de la certificación de la mortalidad en Colombia, 1997-2016. Rev Colomb Cancerol.

[28] Departamento Administrativo Nacional de Estadística (2025). https://www.dane.gov.co/index.php/estadisticas-por-tema/demografia-y-poblacion/proyecciones-de-poblacion.

[29] Ministerio de Salud y Protección Social (2024).

[30] Loos A., Bray F., McCarron P., Weiderpass E., Hakama M., Parkin D. (2004). Sheep and goats: separating cervix and corpus uteri from imprecisely coded uterine cancer deaths, for studies of geographical and temporal variations in mortality. Eur J Cancer.

[31] Departamento Nacional de Planeación (2014).

[32] Segi M. (1960).

[33] Espinosa O., Ramos J., Rojas-Botero M., Fernández-Niño J. (2023). Years of life lost to COVID-19 in 49 countries: a gender- and life cycle-based analysis of the first two years of the pandemic. PLOS Glob Public Health.

[34] Basellini U., Camarda C., Booth H. (2023). Thirty years on: a review of the Lee–Carter method for forecasting mortality. Int J Forecast.

[35] Tosakoon S., Lawrence W., Shiels M., Jackson S. (2024). Sex differences in cancer incidence rates by race and ethnicity: results from the Surveillance, Epidemiology, and End Results (SEER) registry (2000-2019). Cancers (Basel).

[36] Conforti F., Pala L., Bagnardi V. (2018). Cancer immunotherapy efficacy and patients' sex: a systematic review and meta-analysis. Lancet Oncol.

[37] Klein S., Flanagan K. (2016). Sex differences in immune responses. Nat Rev Immunol.

[38] Alfaro T., Martinez-Folgar K., Stern D. (2025). Variability and social patterning of cancer mortality in 343 Latin American cities: an ecological study. Lancet Glob Heal.

[39] Vera R., Juan-Vidal O., Safont-Aguilera M., de la Peña F., del Alba A. (2023). Sex differences in the diagnosis, treatment and prognosis of cancer: the rationale for an individualised approach. Clin Transl Oncol.

[40] Brustugun O., Møller B., Helland Å. (2014). Years of life lost as a measure of cancer burden on a national level. Br J Cancer.

[41] de Vries E., Gallego A., Gil F. (2025). Trends in cancer mortality in the elderly and oldest old in South America. Cancer Epidemiol.

[42] Brisson M., Kim J., Canfell K. (2020). Impact of HPV vaccination and cervical screening on cervical cancer elimination: a comparative modelling analysis in 78 low-income and lower-middle-income countries. Lancet.

[43] Goldin C., Lleras-Muney A. (2019). XX > XY?: the changing female advantage in life expectancy. J Health Econ.

[44] Hernández-Vargas J., Ramírez-Barbosa P., Valbuena-Garcia A., Acuña L., González-Diaz J. (2021). Factors associated with delays in time to treatment initiation in Colombian women with cervical cancer: a cross-sectional analysis. Gynecol Oncol Rep.

[45] Mejia E., Lewis A., Garcés-Palacio I., Hernandez D., Chamberlain R., Soliman A. (2024). Relationship between universal health insurance benefits and prostate cancer mortality in Colombia. BMC Public Health.

[46] Castro-Prieto P., Spijker J., Recaño J. (2024). A quasi-cohort trend analysis of adult obesity in Colombia. J Biosoc Sci.

[47] de Vries E., Amador J., Rincon C., Uribe C., Parkin D. (2017). Cutaneous melanoma attributable to solar radiation in Cali, Colombia. Int J Cancer.

[48] Cordoba-Sanchez V., Lemos M., Tamayo-Lopera D., Sheinfeld Gorin S. (2022). HPV-vaccine hesitancy in Colombia: a mixed-methods study. Vaccines.

[49] Chandanos E., Lagergren J. (2009). The mystery of male dominance in oesophageal cancer and the potential protective role of oestrogen. Eur J Cancer.

[50] Torres-Roman J., Alvarez C., Guerra-Canchari P. (2022). Sex and age differences in mortality trends of gastric cancer among Hispanic/Latino populations in the United States, Latin America, and the Caribbean. Lancet Reg Health Am.

[51] Vargas I., Mogollón-Pérez A., Eguiguren P. (2025). Understanding the health system drivers of delayed cancer diagnosis in public healthcare networks of Chile, Colombia and Ecuador: a qualitative study with health professionals, managers and policymakers. Soc Sci Med.

[52] Reitsma M., Fullman N., Ng M. (2017). Smoking prevalence and attributable disease burden in 195 countries and territories, 1990–2015: a systematic analysis from the Global Burden of Disease Study 2015. Lancet.

[53] Griswold M., Fullman N., Hawley C. (2018). Alcohol use and burden for 195 countries and territories, 1990–2016: a systematic analysis for the Global Burden of Disease Study 2016. Lancet.

[54] Velasco S., Bernal O., Salazar A., Romero J., Moreno Á., Díaz X. (2014). Disponibilidad de servicios de mamografía en Colombia. Rev Colomb Cancerol.

[55] Eibl G., Cruz-Monserrate Z., Korc M. (2018). Diabetes mellitus and obesity as risk factors for pancreatic cancer. J Acad Nutr Diet.

[56] Allemani C., Matsuda T., Di Carlo V. (2018). Global surveillance of trends in cancer survival 2000–14 (CONCORD-3): analysis of individual records for 37,513,025 patients diagnosed with one of 18 cancers from 322 population-based registries in 71 countries. Lancet.

[57] Mosquera-Zamudio A., Gomez-Suarez M., Sprockel J. (2024). Globalization of a telepathology network with artificial intelligence applications in Colombia: the GLORIA program study protocol. J Pathol Inform.

[58] Espinosa O., Friebel R., Bejarano V., García-Ruiz J. (2025). Estimating inequalities in excess mortality and years of potential life lost by health conditions across ethnic minorities in Colombia: a population-based study, 2018-2022. BMJ Open.

