# Supplementary material for: Patterns of sex differences in cancer mortality in Colombia: a population-based analysis, 1980–2023
Source: Lancet Reg Health Am. 2026 Mar 30;58:101465. doi: 10.1016/j.lana.2026.101465 (PMC13066952; doi:10.1016/j.lana.2026.101465)
Supplement: Appendix [file mmc1.docx]

**Patterns of sex differences in cancer mortality in Colombia: a population-based analysis, 1980-2023**

Appendix

| Supplementary Table 1. Classification of causes of deaths by type of cancer according to the International Classification of Diseases | ….…………………………… | 1 |
| --- | --- | --- |
| Supplementary Table 2. Life expectancy at birth of Colombia between 1980 and 2023 | ….…………………………… | 2 |
| Supplementary Table 3. Mortality rate ratio of male compared to female of Colombia, by type of cancer, for each decade between the 1980s and the 2020s | ….…………………………… | 3 |
| Supplementary Figure 1. Age-standardised mortality rate (per 100,000 pop.), stratified by type of cancer, health insurance scheme, and sex, in Colombia, 2012-2023 | ….…………………………… | 5 |
| Supplementary Figure 2. Standardised cancer mortality rates in males and females in Colombia (per 100,000 pop.), by age-group, 1980-2023 | ….…………………………… | 6 |
| Supplementary Figure 3. Percentage of deaths by age-groups in Colombian females, according to type of cancer, 1980-2023 | ….…………………………… | 7 |
| Supplementary Figure 4. Percentage of deaths by age-groups in Colombian males, according to type of cancer, 1980-2023 | ….…………………………… | 8 |
| Supplementary Figure 5. Maps of standardised cancer mortality rates in the departments of Colombia, by decade (1980s, 1990s, 2000s, 2010s, 2020s) | ….…………………………… | 9 |
| Supplementary Figure 6. Standardised mortality rates by type of cancer in males and females in Colombia (per 100,000 pop.), by urban and rural area, and age-group, 1985-2023. (a) 20-29 years old. (b) 30-39 years old. (c) 40-49 years old. (d) 50-59 years old. (e) 60-69 years old. (f) 70-79 years old. (g) 80+ years old | ….…………………………… | 10 |

Supplementary Table 1. Classification of causes of deaths by type of cancer according to the International Classification of Diseases

| **Cause of death** | **9th version 1977** | **10th version 2019** |
| --- | --- | --- |
|  |  |  |
| All neoplasms | 140-175, 179-239 | C00-D48 |
| Bladder | 188 | C67 |
| Breast | 174 | C50 |
| Cervical | 180 | C53 |
| Colon, rectum and anal canal | 153-154 | C18-C21 |
| Oesophagus | 150 | C15 |
| Leukaemia | 204-208 | C91-C95 |
| Liver | 155.0 | C22 |
| Ovary | 183 | C56 |
| Pancreas | 157 | C25 |
| Prostate | 185 | C61 |
| Skin | 172 | C43 |
| Stomach | 151 | C16 |
| Trachea, bronchus, lung | 162 | C33-C34 |
| Uterine | 179*, 182 | C54-C55* |

**Notes:** ICD-10 codes C00–C97 cover malignant neoplasms (cancers), whereas the range C00–D48 also includes benign, in situ, and neoplasms of uncertain or unknown behaviour.

Although ICD-11 was officially presented in 2019, its adoption in Colombia was formally established by the Ministry of Health and Social Protection through Resolution 1442 of 2024, which includes a 12-month transition period. Consequently, ICD-11 was not in operational use for mortality registration during the study period from 2019 to 2023.

* Reallocation of these unspecified causes to cervical cancer were made following international standards.

**Supplementary Table 2. Life expectancy at birth of Colombia between 1980 and 2023**

| **Year** | **Life expectancy at birth** | **Year** | **Life expectancy at birth** | **Year** | **Life expectancy at birth** | **Year** | **Life expectancy at birth** | **Year** | **Life expectancy at birth** |
| --- | --- | --- | --- | --- | --- | --- | --- | --- | --- |
| 1980 | 74·79 | 1990 | 75·60 | 2000 | 75·51 | 2010 | 79·01 | 2020 | 77·95 |
| 1981 | 72·80 | 1991 | 74·80 | 2001 | 75·53 | 2011 | 79·82 | 2021 | 75·50 |
| 1982 | 73·90 | 1992 | 74·76 | 2002 | 75·90 | 2012 | 79·97 | 2022 | 78·87 |
| 1983 | 73·46 | 1993 | 74·97 | 2003 | 76·23 | 2013 | 80·15 | 2023 | 80·05 |
| 1984 | 74·72 | 1994 | 75·22 | 2004 | 76·96 | 2014 | 80·02 |  |  |
| 1985 | 75·78 | 1995 | 75·25 | 2005 | 77·36 | 2015 | 79·78 |  |  |
| 1986 | 76·90 | 1996 | 75·10 | 2006 | 77·54 | 2016 | 80·00 |  |  |
| 1987 | 75·91 | 1997 | 75·85 | 2007 | 77·94 | 2017 | 80·12 |  |  |
| 1988 | 75·60 | 1998 | 76·37 | 2008 | 78·23 | 2018 | 80·38 |  |  |
| 1989 | 75·54 | 1999 | 75·45 | 2009 | 78·82 | 2019 | 80·26 |  |  |
| **1980s** | **75·04** | **1990s** | **75·36** | **2000s** | **77·05** | **2010s** | **79·96** | **2020s** | **78·04** |

**Supplementary Table 3. Mortality rate ratio of male compared to female of Colombia, by type of cancer, for each decade between the 1980s and the 2020s**

| **Age-group** | **Type of cancer** | ***MRR*** | | | | |
| --- | --- | --- | --- | --- | --- | --- |
|  |  | ***1980s*** | ***1990s*** | ***2000s*** | **2010s** | ***2020s*** |
|  |  |  |  |  |  |  |
| ***All ages (20+)*** | All neoplasms | 0·90 (0·89, 0·91) | 1·01 (1·01, 1·01) | 1·07 (1·06, 1·08) | 1·11 (1·10, 1·13) | 1·11 (1·10, 1·12) |
|  | Bladder | 2·05 (0·95, 4·40) | 2·12 (0·94, 4·79) | 2·09 (0·97, 4·52) | 2·44 (0·91, 6·57) | 2·64 (0·84, 8·27) |
|  | Colon, rectum and anal canal | 0·80 (0·71, 0·90) | 0·88 (0·83, 0·93) | 0·93 (0·90, 0·96) | 1·09 (1·05, 1·13) | 1·14 (1·08, 1·19) |
|  | Oesophagus | 1·64 (1·21, 2·21) | 1·89 (1·23, 2·89) | 2·28 (1·21, 4·27) | 2·67 (1·06, 6·72) | 2·83 (0·94, 8·54) |
|  | Leukaemia | 1·15 (1·05, 1·27) | 1·17 (1·06, 1·29) | 1·22 (1·08, 1·37) | 1·28 (1·10, 1·47) | 1·36 (1·12, 1·66) |
|  | Liver | 0·94 (0·89, 0·99) | 0·98 (0·97, 0·99) | 0·98 (0·98, 0·99) | 1·17 (1·07, 1·27) | 1·34 (1·14, 1·59) |
|  | Pancreas | 0·93 (0·90, 0·97) | 0·92 (0·87, 0·96) | 0·95 (0·92, 0·98) | 1·07 (1·03, 1·11) | 1·10 (1·05, 1·16) |
|  | Skin | 1·06 (0·97, 1·15) | 1·15 (0·92, 1·44) | 1·28 (0·90, 1·83) | 1·32 (0·93, 1·87) | 1·36 (0·91, 2·04) |
|  | Stomach | 1·34 (1·25, 1·44) | 1·53 (1·37, 1·72) | 1·73 (1·47, 2·02) | 1·92 (1·55, 2·38) | 1·86 (1·49, 2·31) |
|  | Trachea, bronchus, lung | 1·86 (1·49, 2·33) | 1·79 (1·48, 2·18) | 1·77 (1·47, 2·13) | 1·68 (1·40, 2·01) | 1·61 (1·34, 1·93) |
|  |  |  |  |  |  |  |
| **20-29 years old** | All neoplasms | 1·08 (1·08, 1·08) | 1·17 (1·17, 1·17) | 1·12 (1·12, 1·12) | 1·19 (1·19, 1·20) | 1·30 (1·29, 1·31) |
|  | Bladder | 6·25 (1·96, 19·94) | 2·44 (1·65, 3·61) | 1·03 (1·03, 1·03) | 1·00 (1·00, 1·00) | 2·00 (1·12, 3·54) |
|  | Colon, rectum and anal canal | 1·37 (1·34, 1·41) | 1·10 (1·09, 1·10) | 1·01 (1·01, 1·01) | 1·18 (1·16, 1·19) | 1·18 (1·17, 1·20) |
|  | Oesophagus | 2·00 (1·72, 2·33) | 0·87 (0·83, 0·92) | 2·75 (2·02, 3·75) | 1·43 (1·27, 1·62) | 2·49 (1·50, 4·15) |
|  | Leukaemia | 1·36 (1·34, 1·37) | 1·44 (1·42, 1·45) | 1·46 (1·45, 1·48) | 1·51 (1·49, 1·53) | 1·57 (1·53, 1·61) |
|  | Liver | 1·54 (1·43, 1·66) | 2·30 (2·03, 2·61) | 1·37 (1·33, 1·42) | 1·10 (1·08, 1·11) | 2·08 (1·75, 2·47) |
|  | Pancreas | 2·01 (1·75, 2·32) | 1·49 (1·39, 1·60) | 1·08 (1·07, 1·10) | 1·60 (1·46, 1·74) | 1·20 (1·15, 1·25) |
|  | Skin | 1·04 (1·04, 1·04) | 0·61 (0·53, 0·70) | 1·12 (1·10, 1·14) | 1·23 (1·18, 1·27) | 2·14 (1·69, 2·70) |
|  | Stomach | 0·96 (0·96, 0·97) | 1·29 (1·27, 1·31) | 1·02 (1·02, 1·02) | 0·98 (0·98, 0·98) | 1·10 (1·09, 1·11) |
|  | Trachea, bronchus, lung | 1·70 (1·61, 1·79) | 1·49 (1·44, 1·54) | 1·59 (1·53, 1·65) | 1·90 (1·77, 2·03) | 1·13 (1·11, 1·16) |
|  |  |  |  |  |  |  |
| **30-39 years old** | All neoplasms | 0·62 (0·62, 0·63) | 0·67 (0·67, 0·68) | 0·68 (0·68, 0·69) | 0·68 (0·68, 0·68) | 0·69 (0·69, 0·70) |
|  | Bladder | 1·04 (1·04, 1·04) | 2·46 (2·02, 3·00) | 1·91 (1·67, 2·20) | 2·27 (1·93, 2·66) | 1·92 (1·58, 2·34) |
|  | Colon, rectum and anal canal | 0·87 (0·86, 0·88) | 1·11 (1·11, 1·12) | 0·90 (0·89, 0·91) | 0·94 (0·94, 0·95) | 1·06 (1·06, 1·07) |
|  | Oesophagus | 1·24 (1·21, 1·27) | 1·89 (1·74, 2·04) | 1·74 (1·63, 1·85) | 3·11 (2·48, 3·89) | 1·94 (1·61, 2·34) |
|  | Leukaemia | 1·08 (1·08, 1·08) | 1·20 (1·20, 1·21) | 1·23 (1·22, 1·23) | 1·25 (1·25, 1·26) | 1·49 (1·45, 1·52) |
|  | Liver | 1·63 (1·54, 1·73) | 0·84 (0·82, 0·86) | 1·27 (1·26, 1·28) | 1·10 (1·10, 1·10) | 1·34 (1·29, 1·38) |
|  | Pancreas | 1·32 (1·29, 1·36) | 1·49 (1·44, 1·54) | 1·23 (1·22, 1·25) | 1·31 (1·28, 1·33) | 1·09 (1·08, 1·10) |
|  | Skin | 0·58 (0·51, 0·64) | 0·76 (0·72, 0·80) | 1·37 (1·33, 1·41) | 0·75 (0·72, 0·78) | 0·89 (0·87, 0·91) |
|  | Stomach | 1·30 (1·29, 1·32) | 1·33 (1·32, 1·34) | 1·17 (1·16, 1·17) | 0·99 (0·99, 1·00) | 1·01 (1·01, 1·01) |
|  | Trachea, bronchus, lung | 1·45 (1·41, 1·48) | 1·16 (1·15, 1·16) | 1·31 (1·30, 1·33) | 1·17 (1·17, 1·18) | 1·34 (1·31, 1·38) |
|  |  |  |  |  |  |  |
| **40-49 years old** | All neoplasms | 0·55 (0·55, 0·56) | 0·63 (0·62, 0·63) | 0·68 (0·67, 0·68) | 0·66 (0·65, 0·66) | 0·62 (0·62, 0·63) |
|  | Bladder | 1·78 (1·62, 1·96) | 2·03 (1·84, 2·23) | 1·58 (1·51, 1·64) | 1·49 (1·44, 1·55) | 1·63 (1·50, 1·77) |
|  | Colon, rectum and anal canal | 0·70 (0·69, 0·72) | 0·88 (0·88, 0·89) | 0·94 (0·93, 0·94) | 0·97 (0·97, 0·97) | 1·03 (1·03, 1·03) |
|  | Oesophagus | 1·64 (1·57, 1·71) | 2·21 (2·08, 2·34) | 2·28 (2·15, 2·42) | 1·94 (1·85, 2·03) | 1·61 (1·51, 1·72) |
|  | Leukaemia | 0·96 (0·95, 0·96) | 1·16 (1·15, 1·16) | 1·25 (1·25, 1·26) | 1·18 (1·18, 1·18) | 1·33 (1·31, 1·34) |
|  | Liver | 0·98 (0·98, 0·98) | 1·04 (1·04, 1·04) | 1·13 (1·13, 1·13) | 1·02 (1·01, 1·02) | 1·26 (1·25, 1·28) |
|  | Pancreas | 0·94 (0·94, 0·94) | 1·19 (1·19, 1·20) | 1·32 (1·31, 1·33) | 1·38 (1·37, 1·39) | 1·21 (1·20, 1·22) |
|  | Skin | 0·92 (0·91, 0·93) | 1·35 (1·31, 1·39) | 1·36 (1·33, 1·38) | 1·23 (1·23, 1·24) | 1·16 (1·15, 1·17) |
|  | Stomach | 1·40 (1·39, 1·42) | 1·64 (1·62, 1·65) | 1·69 (1·67, 1·70) | 1·53 (1·52, 1·55) | 1·25 (1·24, 1·25) |
|  | Trachea, bronchus, lung | 1·40 (1·38, 1·42) | 1·38 (1·37, 1·39) | 1·48 (1·46, 1·49) | 1·01 (1·01, 1·02) | 1·08 (1·08, 1·08) |
|  |  |  |  |  |  |  |
| **50-59 years old** | All neoplasms | 0·72 (0·72, 0·73) | 0·78 (0·77, 0·78) | 0·85 (0·85, 0·86) | 0·87 (0·87, 0·87) | 0·79 (0·79, 0·79) |
|  | Bladder | 1·54 (1·46, 1·61) | 2·15 (2·00, 2·31) | 2·39 (2·25, 2·54) | 1·95 (1·88, 2·03) | 1·56 (1·51, 1·62) |
|  | Colon, rectum and anal canal | 0·78 (0·77, 0·78) | 0·87 (0·86, 0·87) | 0·95 (0·95, 0·95) | 1·05 (1·05, 1·06) | 0·97 (0·96, 0·97) |
|  | Oesophagus | 1·83 (1·77, 1·89) | 1·97 (1·90, 2·04) | 3·06 (2·90, 3·23) | 2·80 (2·68, 2·93) | 2·76 (2·54, 2·99) |
|  | Leukaemia | 1·07 (1·06, 1·08) | 1·00 (1·00, 1·00) | 1·06 (1·05, 1·06) | 1·16 (1·15, 1·16) | 1·04 (1·04, 1·04) |
|  | Liver | 0·94 (0·94, 0·94) | 0·96 (0·96, 0·97) | 1·05 (1·05, 1·05) | 1·19 (1·19, 1·20) | 1·20 (1·20, 1·21) |
|  | Pancreas | 1·01 (1·00, 1·01) | 0·94 (0·94, 0·94) | 1·11 (1·11, 1·11) | 1·24 (1·24, 1·25) | 1·16 (1·16, 1·16) |
|  | Skin | 1·05 (1·03, 1·06) | 1·43 (1·38, 1·48) | 1·42 (1·39, 1·45) | 1·32 (1·31, 1·33) | 1·23 (1·22, 1·24) |
|  | Stomach | 1·50 (1·48, 1·51) | 1·73 (1·71, 1·74) | 2·01 (1·99, 2·03) | 2·11 (2·09, 2·13) | 1·74 (1·73, 1·76) |
|  | Trachea, bronchus, lung | 1·62 (1·60, 1·65) | 1·58 (1·57, 1·60) | 1·76 (1·74, 1·77) | 1·48 (1·47, 1·49) | 1·18 (1·18, 1·18) |
|  |  |  |  |  |  |  |
| **60-69 years old** | All neoplasms | 0·95 (0·95, 0·95) | 1·00 (1·00, 1·00) | 1·08 (1·08, 1·08) | 1·19 (1·19, 1·19) | 1·19 (1·19, 1·19) |
|  | Bladder | 1·73 (1·66, 1·80) | 1·89 (1·82, 1·97) | 2·25 (2·16, 2·34) | 2·76 (2·65, 2·87) | 3·09 (2·89, 3·31) |
|  | Colon, rectum and anal canal | 0·80 (0·80, 0·81) | 0·85 (0·85, 0·86) | 0·95 (0·95, 0·96) | 1·24 (1·24, 1·25) | 1·25 (1·25, 1·25) |
|  | Oesophagus | 1·68 (1·65, 1·72) | 1·96 (1·92, 2·01) | 2·33 (2·27, 2·40) | 3·28 (3·16, 3·42) | 3·28 (3·09, 3·49) |
|  | Leukaemia | 1·07 (1·07, 1·08) | 1·12 (1·12, 1·13) | 1·11 (1·11, 1·12) | 1·18 (1·18, 1·18) | 1·17 (1·17, 1·17) |
|  | Liver | 0·87 (0·87, 0·88) | 0·98 (0·98, 0·98) | 0·98 (0·98, 0·99) | 1·40 (1·39, 1·40) | 1·56 (1·55, 1·58) |
|  | Pancreas | 0·85 (0·85, 0·86) | 0·89 (0·89, 0·90) | 0·99 (0·99, 0·99) | 1·12 (1·12, 1·12) | 1·25 (1·25, 1·26) |
|  | Skin | 1·11 (1·09, 1·13) | 1·05 (1·05, 1·05) | 1·37 (1·35, 1·39) | 1·35 (1·34, 1·36) | 1·69 (1·65, 1·74) |
|  | Stomach | 1·44 (1·43, 1·45) | 1·55 (1·54, 1·56) | 1·95 (1·93, 1·97) | 2·39 (2·36, 2·41) | 2·35 (2·32, 2·39) |
|  | Trachea, bronchus, lung | 1·98 (1·95, 2·01) | 1·78 (1·76, 1·80) | 1·80 (1·79, 1·81) | 1·86 (1·85, 1·87) | 1·77 (1·76, 1·79) |
|  |  |  |  |  |  |  |
| **70-79 years old** | All neoplasms | 1·09 (1·09, 1·09) | 1·24 (1·24, 1·24) | 1·25 (1·25, 1·25) | 1·34 (1·34, 1·34) | 1·42 (1·42, 1·42) |
|  | Bladder | 2·49 (2·36, 2·63) | 2·21 (2·13, 2·29) | 2·05 (2·00, 2·11) | 2·72 (2·64, 2·81) | 2·76 (2·64, 2·88) |
|  | Colon, rectum and anal canal | 0·81 (0·81, 0·82) | 0·89 (0·88, 0·89) | 0·93 (0·92, 0·93) | 1·09 (1·09, 1·09) | 1·25 (1·25, 1·25) |
|  | Oesophagus | 1·59 (1·56, 1·62) | 1·79 (1·76, 1·82) | 2·18 (2·13, 2·23) | 2·75 (2·68, 2·83) | 3·06 (2·92, 3·20) |
|  | Leukaemia | 1·36 (1·33, 1·38) | 1·15 (1·15, 1·16) | 1·23 (1·22, 1·23) | 1·39 (1·38, 1·39) | 1·70 (1·68, 1·72) |
|  | Liver | 0·94 (0·93, 0·94) | 0·96 (0·95, 0·96) | 0·92 (0·92, 0·93) | 1·11 (1·10, 1·11) | 1·32 (1·32, 1·32) |
|  | Pancreas | 0·94 (0·94, 0·94) | 0·88 (0·87, 0·88) | 0·82 (0·81, 0·82) | 0·98 (0·98, 0·98) | 1·02 (1·02, 1·03) |
|  | Skin | 1·11 (1·10, 1·13) | 1·07 (1·07, 1·07) | 1·17 (1·17, 1·18) | 1·46 (1·44, 1·48) | 1·46 (1·44, 1·48) |
|  | Stomach | 1·25 (1·25, 1·26) | 1·56 (1·55, 1·56) | 1·65 (1·64, 1·66) | 2·07 (2·05, 2·08) | 2·26 (2·24, 2·29) |
|  | Trachea, bronchus, lung | 2·09 (2·05, 2·12) | 2·09 (2·06, 2·11) | 1·80 (1·79, 1·81) | 1·76 (1·75, 1·77) | 1·79 (1·78, 1·80) |
|  |  |  |  |  |  |  |
| **80+ years old** | All neoplasms | 1·16 (1·16, 1·16) | 1·36 (1·36, 1·36) | 1·47 (1·47, 1·47) | 1·39 (1·39, 1·39) | 1·45 (1·45, 1·45) |
|  | Bladder | 2·37 (2·27, 2·49) | 2·33 (2·26, 2·41) | 1·97 (1·94, 2·01) | 2·22 (2·18, 2·26) | 2·79 (2·70, 2·88) |
|  | Colon, rectum and anal canal | 0·77 (0·75, 0·78) | 0·87 (0·86, 0·87) | 0·87 (0·86, 0·88) | 0·96 (0·95, 0·96) | 1·04 (1·03, 1·05) |
|  | Oesophagus | 1·44 (1·43, 1·45) | 1·81 (1·79, 1·84) | 2·00 (1·97, 2·03) | 2·00 (1·97, 2·03) | 2·37 (2·31, 2·43) |
|  | Leukaemia | 1·41 (1·40, 1·43) | 1·28 (1·28, 1·28) | 1·41 (1·40, 1·41) | 1·33 (1·33, 1·33) | 1·42 (1·42, 1·42) |
|  | Liver | 0·99 (0·98, 1·01) | 1·00 (0·99, 1·02) | 0·93 (0·92, 0·94) | 0·95 (0·94, 0·95) | 1·15 (1·14, 1·16) |
|  | Pancreas | 1·00 (0·99, 1·01) | 0·85 (0·84, 0·87) | 0·84 (0·83, 0·85) | 0·83 (0·82, 0·84) | 0·85 (0·83, 0·86) |
|  | Skin | 1·15 (1·15, 1·16) | 1·44 (1·43, 1·46) | 1·10 (1·08, 1·11) | 1·33 (1·33, 1·33) | 1·16 (1·14, 1·17) |
|  | Stomach | 1·09 (1·08, 1·09) | 1·28 (1·28, 1·28) | 1·43 (1·43, 1·44) | 1·52 (1·51, 1·52) | 1·67 (1·66, 1·67) |
|  | Trachea, bronchus, lung | 1·91 (1·88, 1·94) | 1·86 (1·84, 1·88) | 1·82 (1·81, 1·84) | 1·59 (1·58, 1·59) | 1·58 (1·58, 1·58) |

Note: *MRR (mortality rate ratio; a number >1 means more mortality in males). 95% CIs in parentheses.

**Supplementary Figure 1. Age-standardised mortality rate (per 100,000 pop.), stratified by type of cancer, health insurance scheme, and sex, in Colombia, 2012-2023**


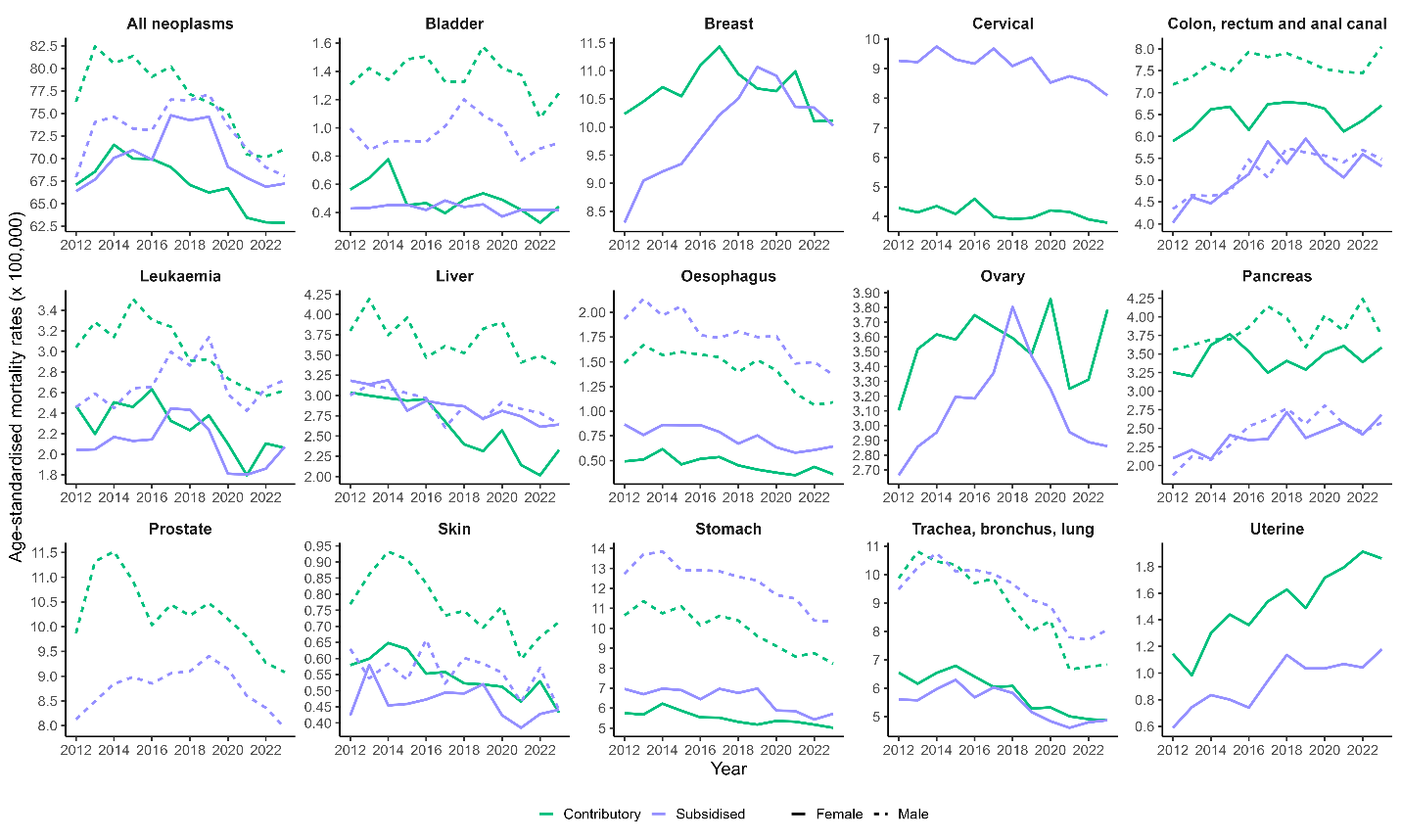


**Supplementary Figure 2. Standardised cancer mortality rates in males and females in Colombia (per 100,000 pop.), by age-group, 1980-2023**


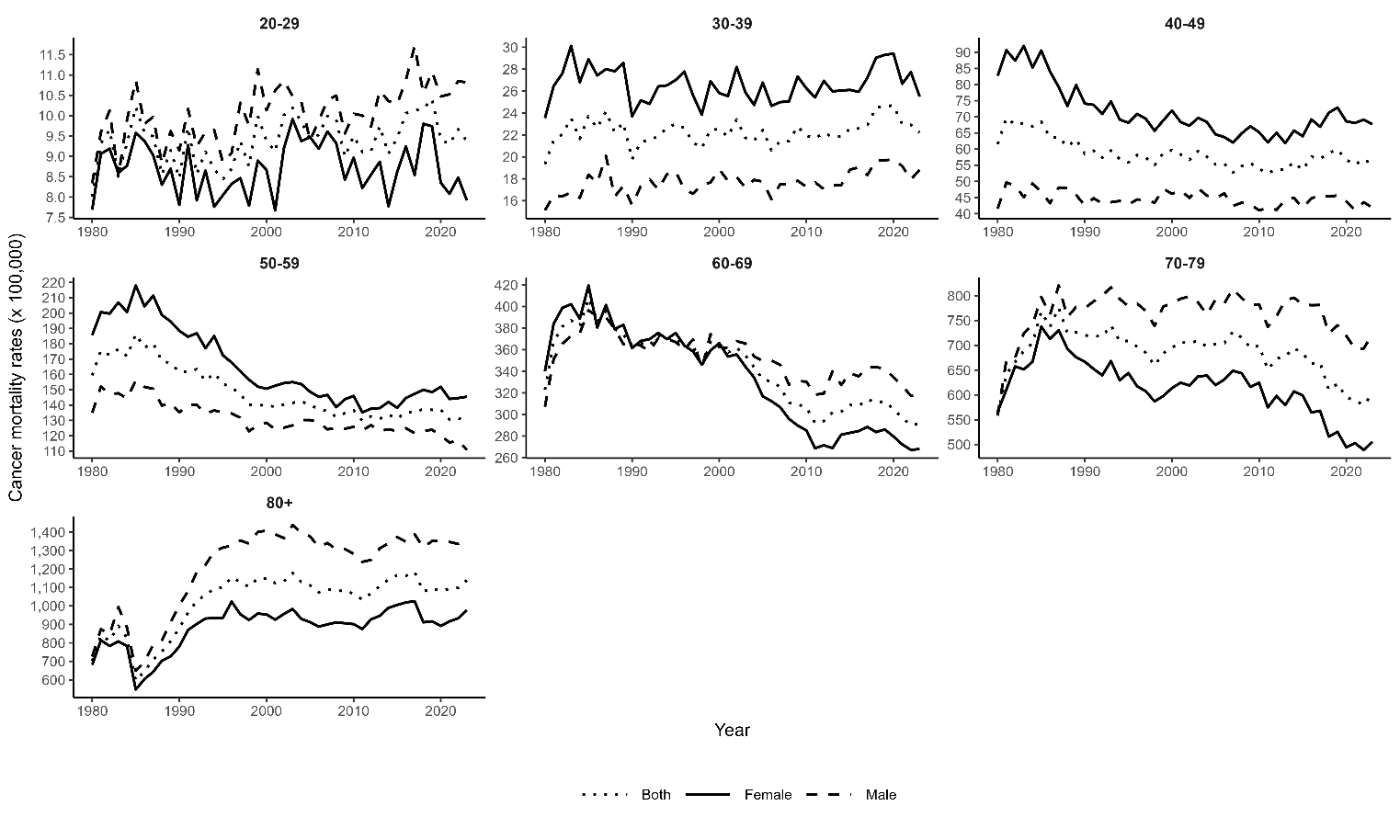


**Supplementary Figure 3. Percentage of deaths by age-groups in Colombian females, according to type of cancer, 1980-2023**


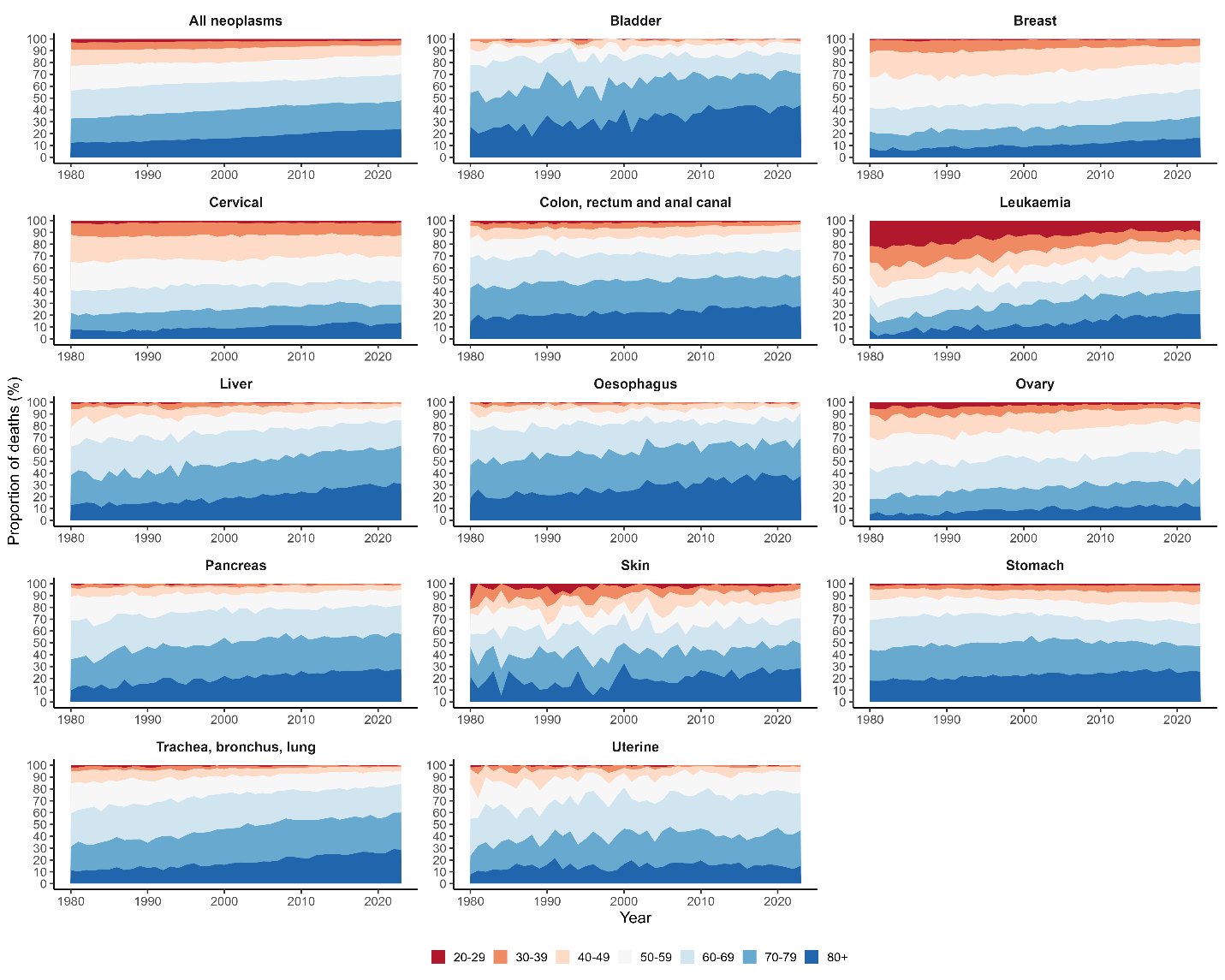


**Supplementary Figure 4. Percentage of deaths by age-groups in Colombian males, according to type of cancer, 1980-2023**


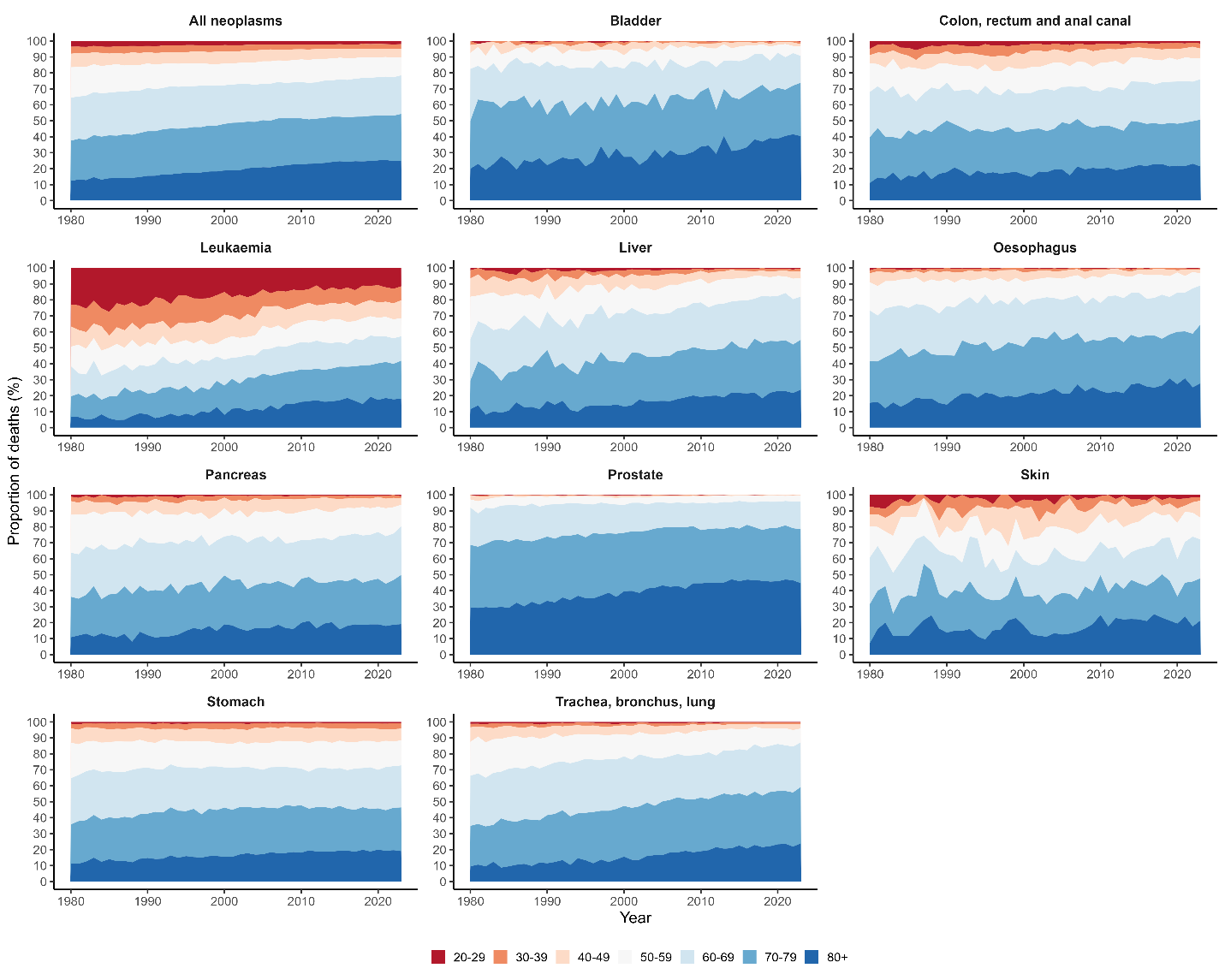


**Supplementary Figure 5. Maps of standardised cancer mortality rates in the departments of Colombia, by decade (1980s, 1990s, 2000s, 2010s, 2020s)**


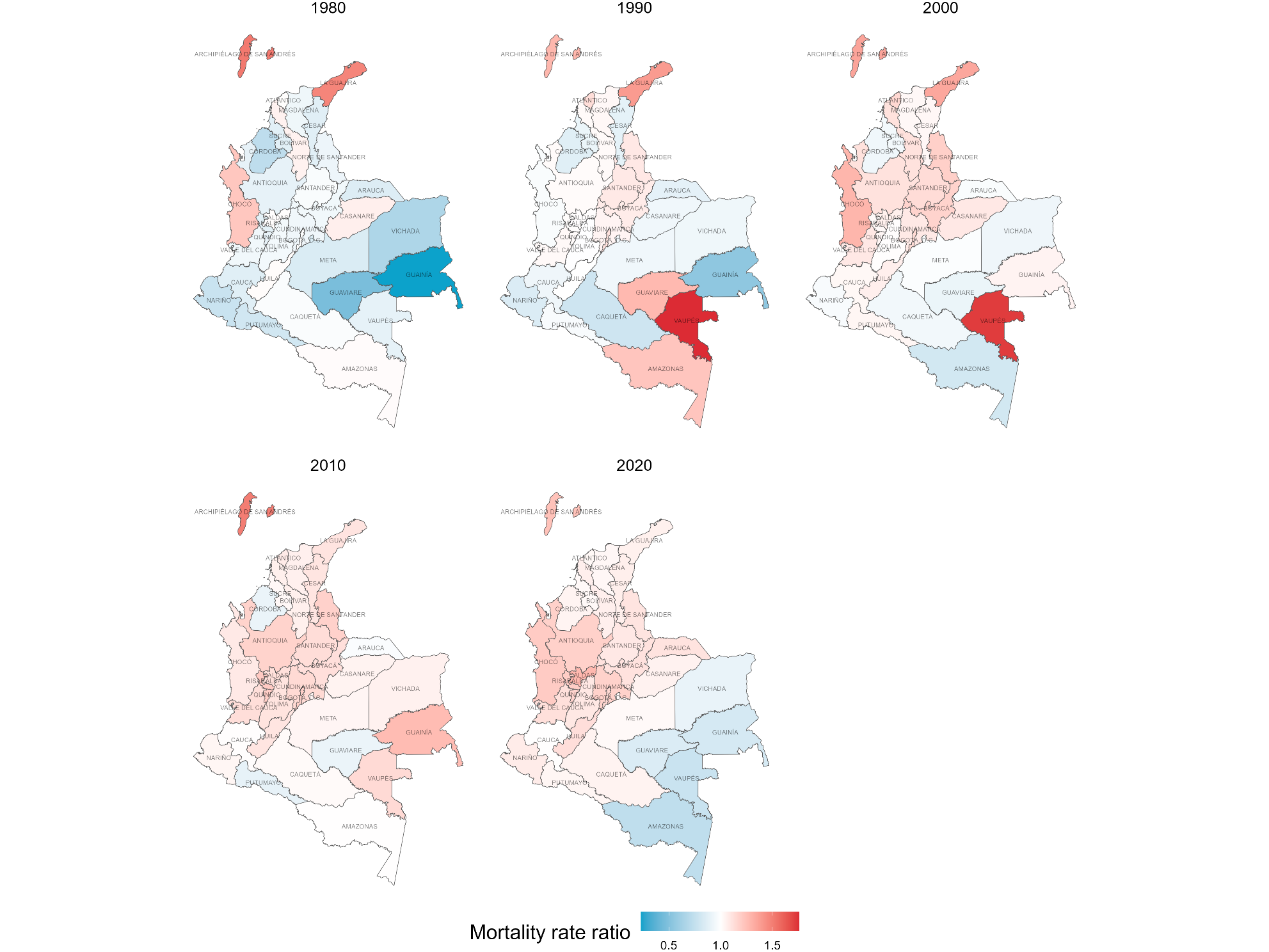


**Supplementary Figure 6. Standardised mortality rates by type of cancer in males and females in Colombia (per 100,000 pop.), by urban and rural area, and age-group, 1985-2023. (a) 20-29 years old. (b) 30-39 years old. (c) 40-49 years old. (d) 50-59 years old. (e) 60-69 years old. (f) 70-79 years old. (g) 80+ years old**

**
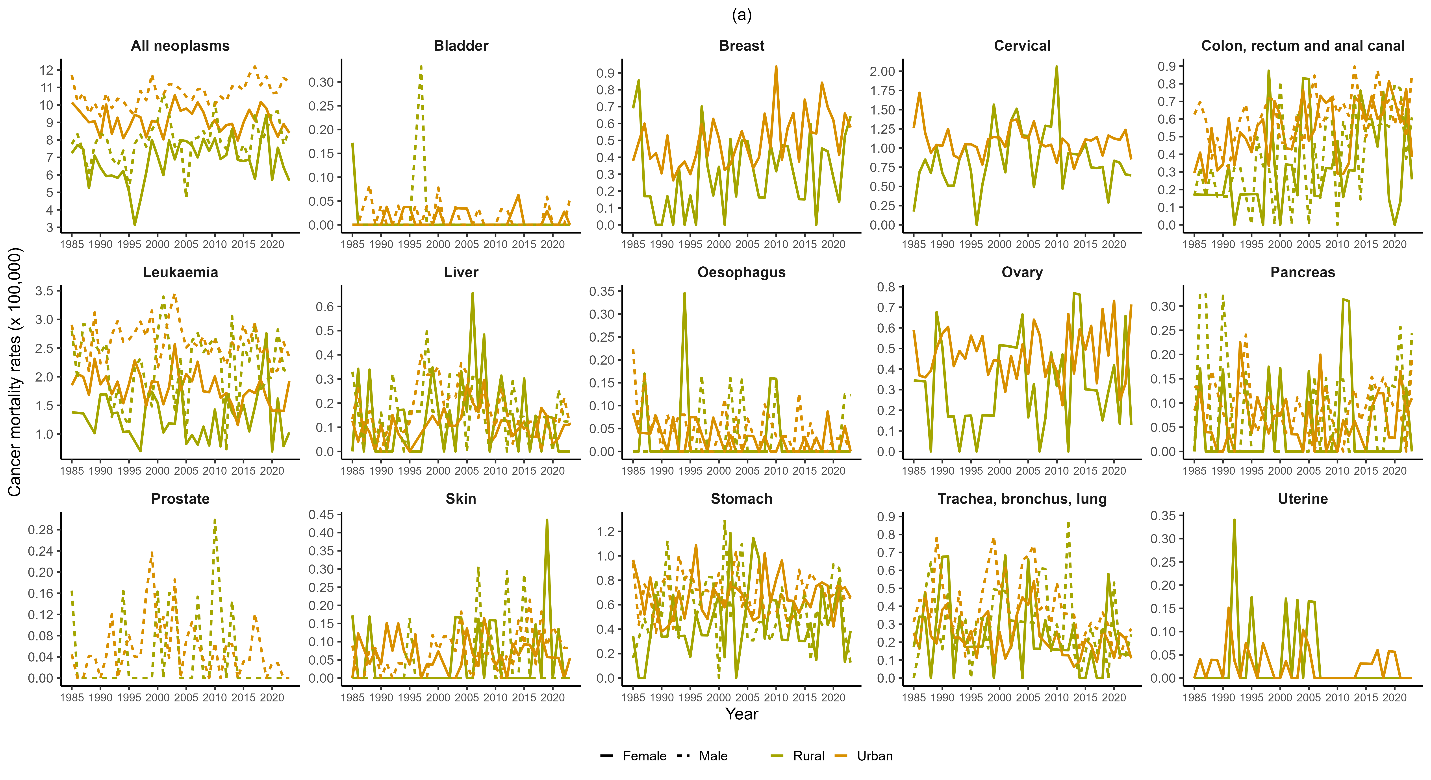
**

**
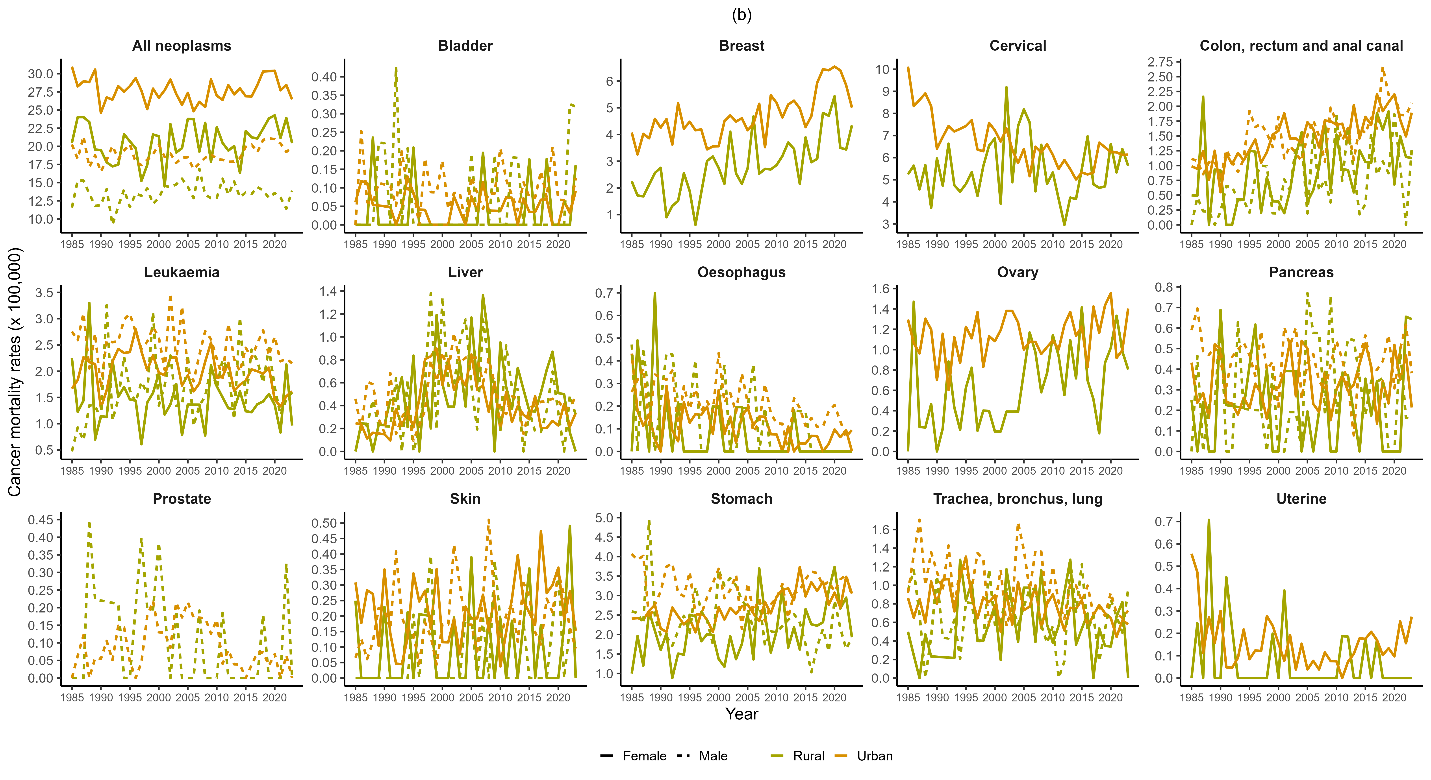
**

**
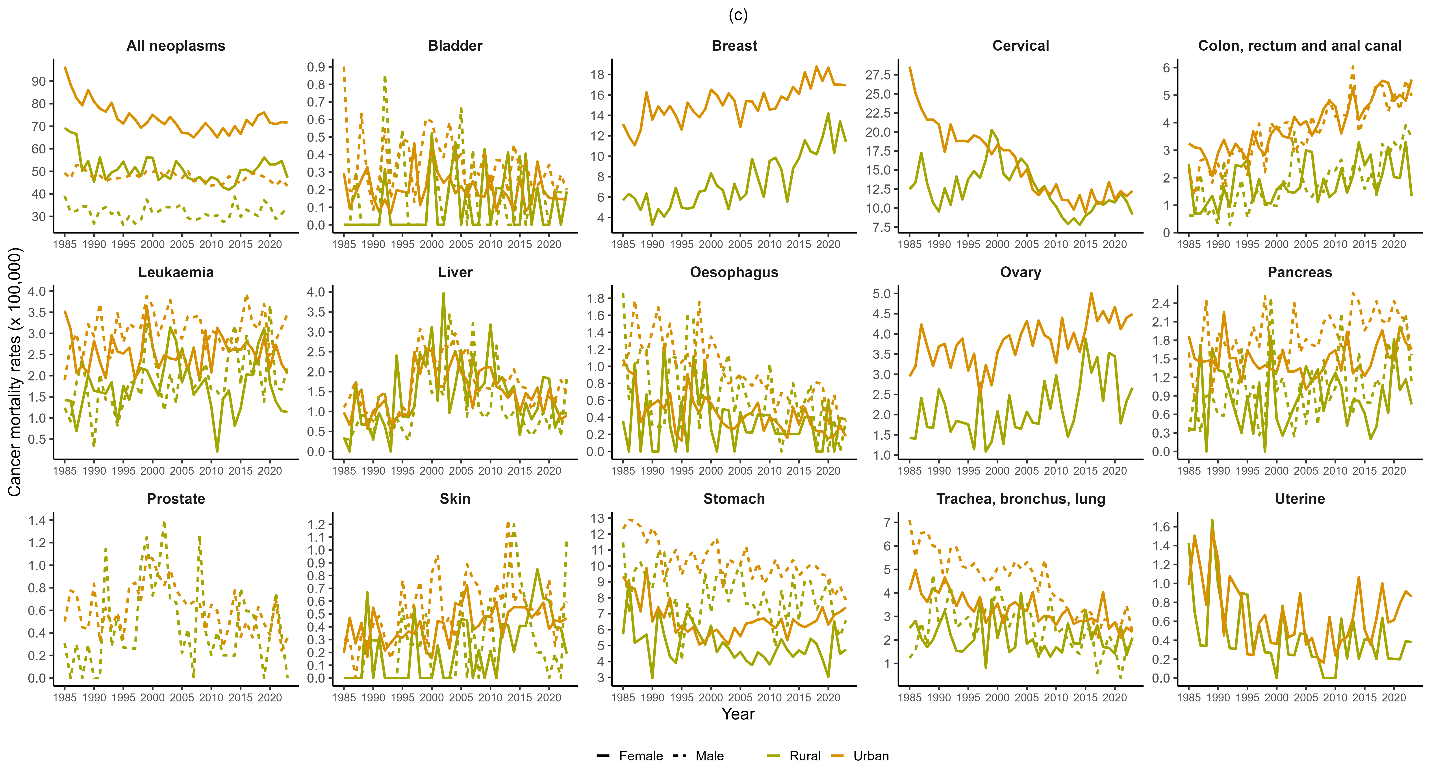
**

**
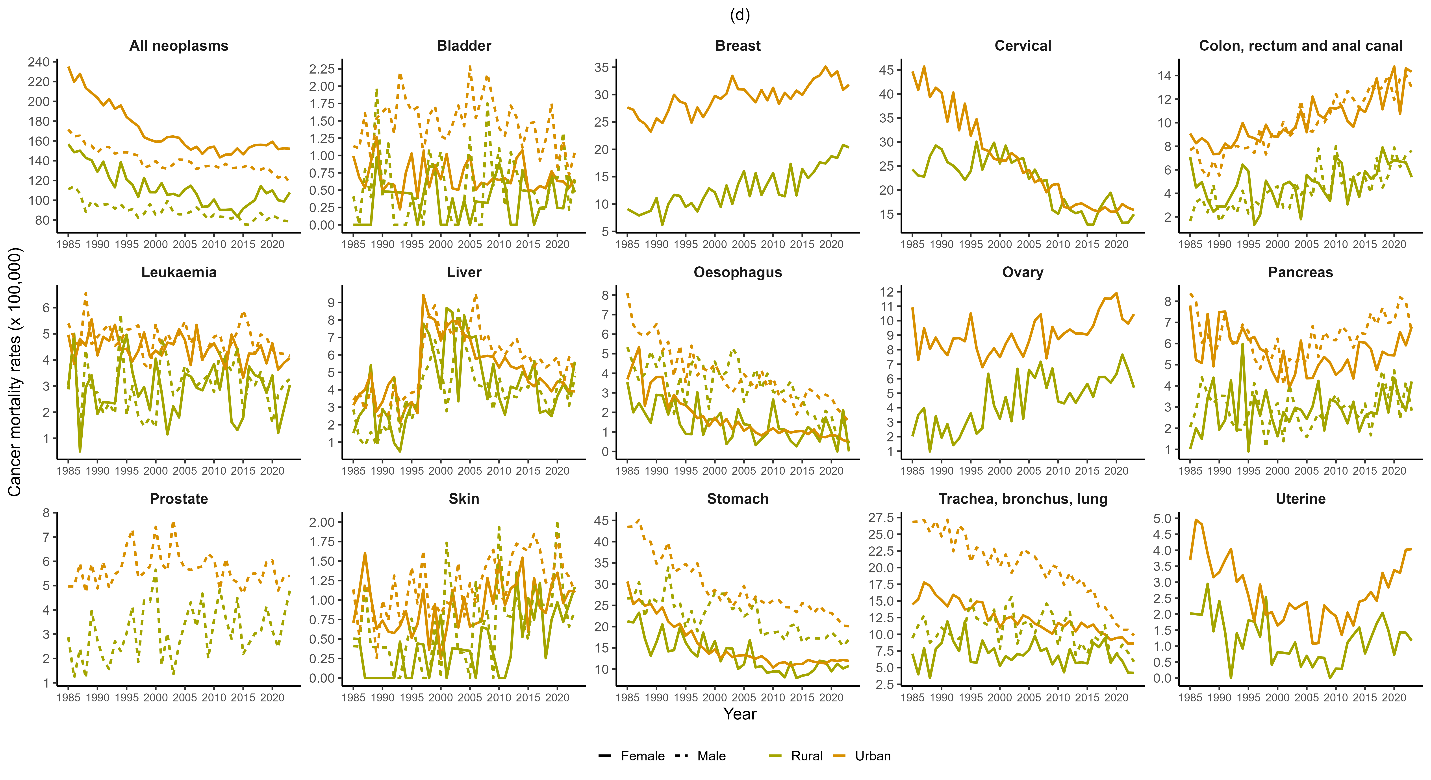
**

**
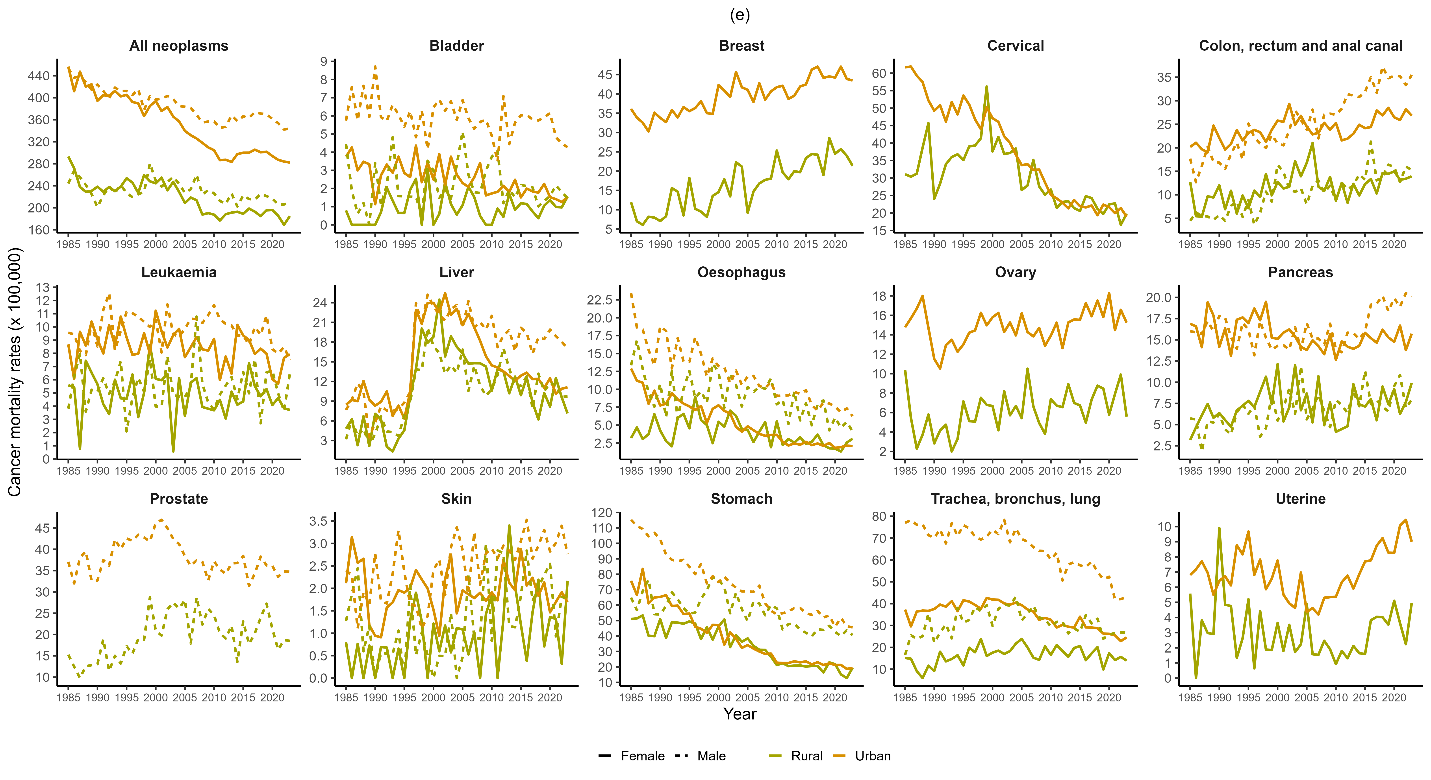
**

**
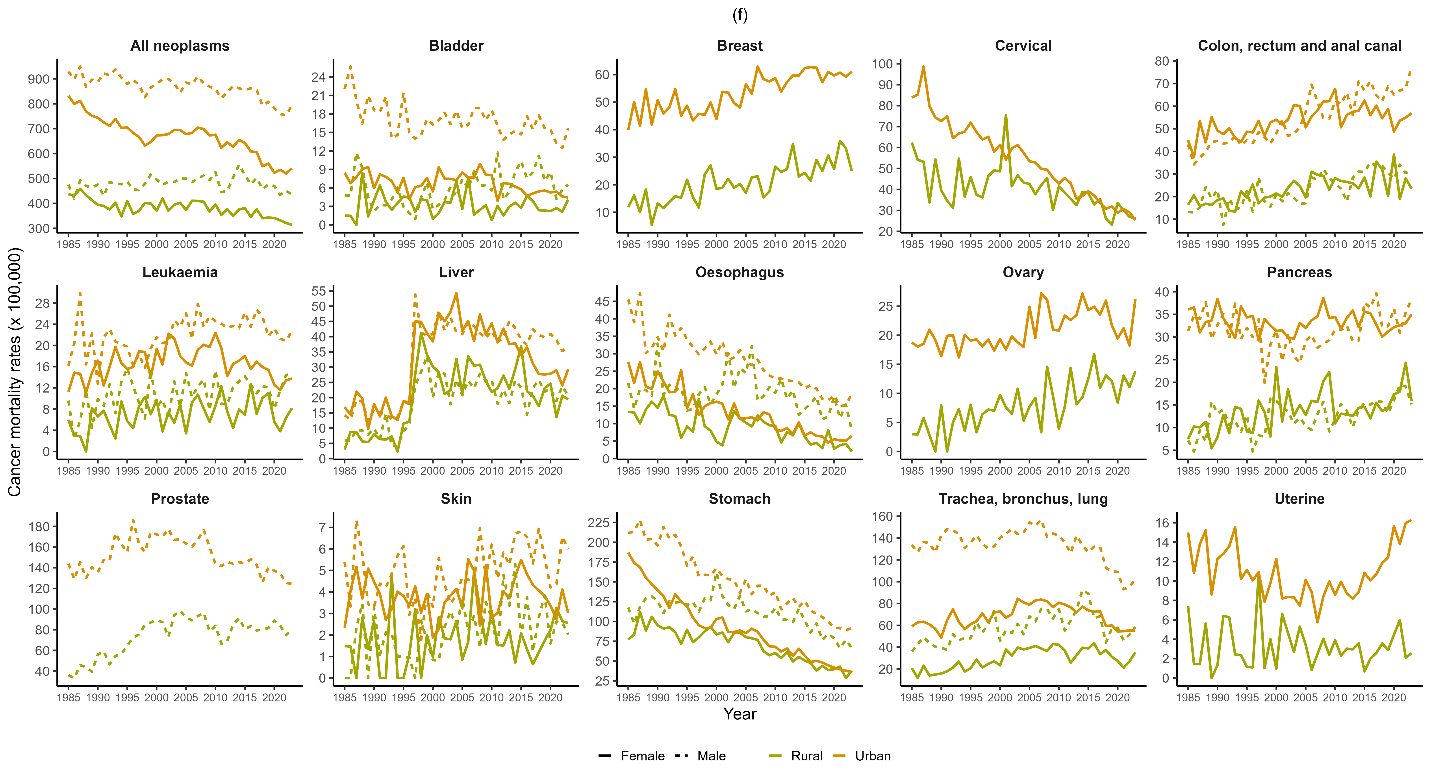
**

**
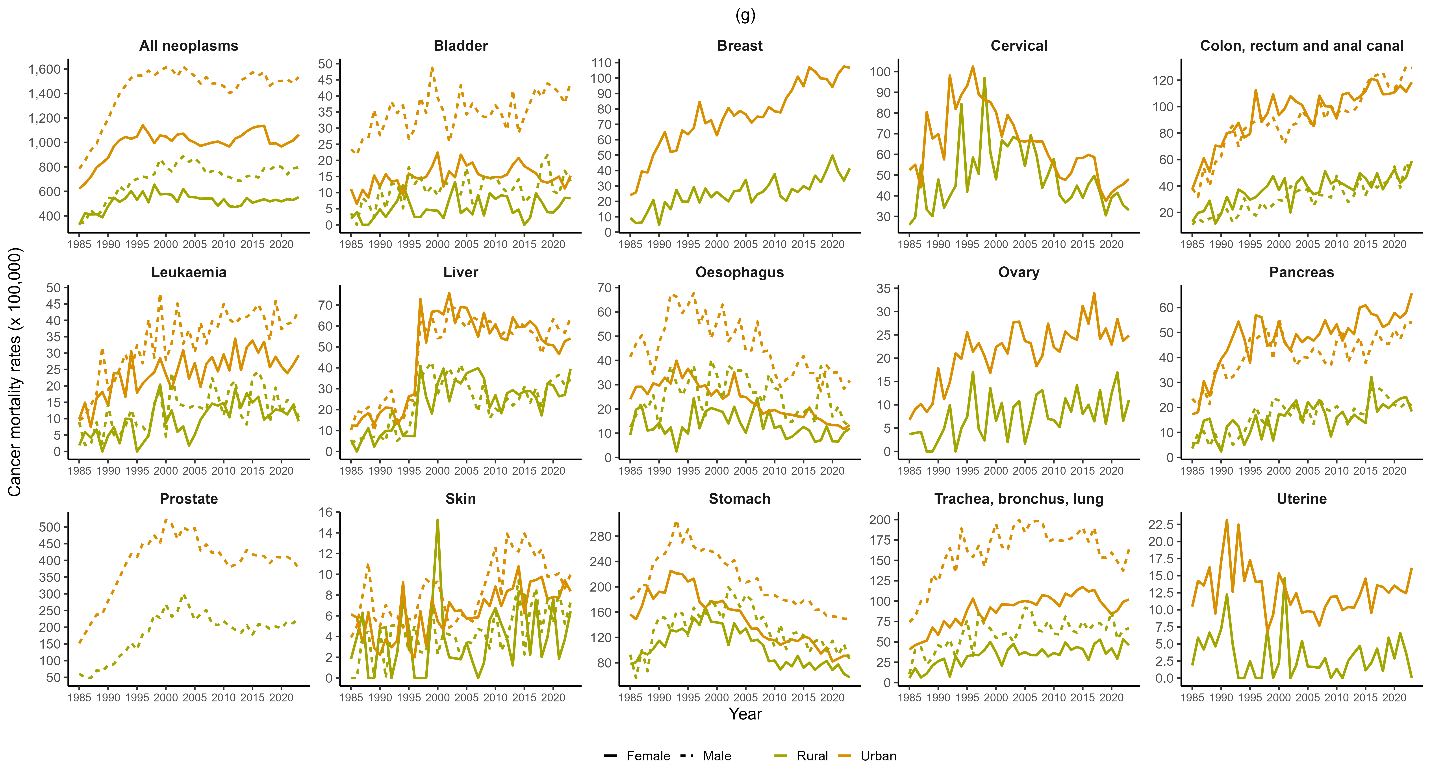
**
